# CardioSynergyNet: A Closed-Loop Multi-Task Deep Learning Architecture for Cardiac Segmentation and Biomarkers with Diagnosis from Paired ED-ES Cine-MRI

**DOI:** 10.3390/tomography12090137

**Published:** 2026-09-21

**Authors:** Saeed Alqahtani

**Affiliations:** 1 Radiological Sciences Department, College of Applied Medical Sciences, Najran University, Najran 61441, Saudi Arabia; salqahtani@nu.edu.sa; 2 Health Research Center, Najran University, Najran 61441, Saudi Arabia

**Keywords:** cardiac MRI segmentation, multi-task deep learning, disease classification, biomarker estimation, cross-phase deformation attention, convolutional neural networks, cardiomyopathy diagnosis

## Abstract

Background: Cardiac MRI analysis typically treats segmentation, biomarker estimation, and disease classification as separate problems, even though clinical biomarkers are themselves derived from segmentation masks and diagnosis depends on both. This paper investigates whether a single jointly trained network can perform all three tasks without sacrificing accuracy on any of them. Methods: I propose CardioSynergyNet, which is a multi-task network with a weight-shared encoder for end-diastolic (ED) and end-systolic (ES) frames. Three modules link the tasks: Cross-Phase Deformation Attention (CPDA) models ED–ES deformation, a Differentiable Biomarker Extraction Layer (DBEL) computes eight clinical biomarkers directly from the soft segmentation mask, and Class-Conditional Prototype Feedback (CCPF) conditions segmentation on the predicted disease class. The model was trained and tested on 150 patients (30 per class across five diagnostic groups), who were split patient-wise into 110/20/20 train/validation/test, yielding 1489 paired ED/ES slices. Results: On the test set, the model achieved a mean ED Dice score of 0.905 (computed across all four classes, including background), with the right ventricle being the hardest structure, particularly at ES. Biomarker regression was accurate for area- and mass-based quantities (R > 0.92) but weaker for ratio-based biomarkers such as ejection fraction (R = 0.66). Disease classification reached 76.65% accuracy with most confusions occurring between clinically similar disease pairs. Conclusions: The joint training of segmentation, biomarker extraction, and classification achieves performance competitive with task-specific models while keeping outputs across tasks consistent with one another, supporting cross-task feedback as a viable direction for integrated cardiac MRI analysis.

## 1. Introduction

Cardiovascular disease continues to be the most frequent cause of death globally, and cardiac magnetic resonance imaging (cine-MRI) is considered the non-invasive standard-of-care for evaluating the structure and function of the ventricles [1,2]. Accurate delineation of the left ventricle (LV), right ventricle (RV), and myocardium throughout the cardiac cycle is essential to allow the computation of clinically relevant parameters such as ejection fraction, myocardial mass, and wall thickness, which are used for diagnosis, risk stratification, and treatment planning in a wide range of cardiac diseases including dilated cardiomyopathy, hypertrophic cardiomyopathy, and myocardial infarction [3,4]. These structures are difficult to manually contour in both the end-diastolic (ED) and end-systolic (ES) phases, are time consuming, and are subject to significant inter- and intra-observer variability, which has led to twenty years of research on automated segmentation methods [5].

The recent advances in deep learning have led to significant progress in this area with recursive convolutional encoder–decoder architectures now regularly performing on par with the agreement of expert clinicians on benchmark datasets such as the corpus of the Automated Cardiac Diagnosis Challenge (ACDC) [6]. MRI and CT systematic reviews show that U-Net-based architectures with attention mechanisms or transformer blocks are at the top of the current state of the art for ventricular and atrial segmentation [1,7]. More extensive benchmarking has been carried out on real-time, free-breathing acquisitions, showing that nnU-Net-style configurations train and generalize well beyond breath-hold cine acquisitions; however, acquisition conditions beyond the training distribution cause performance degradation [3]. In addition to ventricular structures, significant efforts in recent years have been dedicated to left atrial (LA) segmentation using late gadolinium-enhanced (LGE) MRI, which is clinically relevant for planning atrial fibrillation ablation procedures but is also hampered by thin and highly variable atrial wall anatomy and weak tissue contrast [8,9]. Lightweight and edge-aware networks such as Usformer and boundary-enhanced encoder–decoder (BE-ED) designs have been proposed specifically to address these challenges in the LA setting [10,11], and joint segmentation-quantification frameworks such as AtrialJSQnet show that integrating structural delineation with downstream clinical measurement can enhance both tasks [12].

Despite these advances, most current pipelines treat cardiac segmentation [13] and disease classification as distinct tasks, which are performed either sequentially or by separately trained multi-task heads on top of a shared encoder [14,15]. This separation discards a substantial amount of mutually informative signal: pixel-level segmentation outputs encode the precise anatomical evidence that a clinician would use to reach a diagnosis, while the diagnosis itself constrains the plausible space of anatomical configurations that the segmentation should produce. Another limitation concerns the handling of the ED and ES phases. While these two frames are obtained from the same patient and the same anatomical volume, most segmentation networks operate on each frame separately, without accounting for the motion that connects one phase to the next, which also contains diagnostically relevant information [16,17]. Joint registration–segmentation frameworks have begun to address this gap in the wider medical imaging literature, but applications that combine phase-aware deformation modeling with simultaneous diagnostic classification remain comparatively rare [18,19].

In this paper, CardioSynergyNet, a closed-loop multi-task deep learning architecture for joint cardiac segmentation and disease classification from paired ED-ES cine-MRI, is proposed to address these gaps. CardioSynergyNet is built on three coupled modules that create bidirectional information flow between segmentation and classification, which is in contrast to previous multi-task cardiac models that usually share only an encoder backbone between tasks [15,20]. First, a Cross-Phase Deformation Attention (CPDA) module explicitly estimates the ED-to-ES deformation field, building on existing joint motion-segmentation formulations, and routes the resulting fused representation directly into a diagnostic pathway rather than using it only to regularize segmentation. Second, a Differentiable Biomarker Extraction Layer (DBEL), informed by prior work on biomarker-aware segmentation, directly computes clinically interpretable indices (ejection fraction proxy, myocardial mass, wall thickness, chamber area ratios) from soft segmentation probabilities in a fully differentiable manner, avoiding the hard-thresholding step that hinders gradient flow in previous ensemble-based diagnostic pipelines. Third, a Class-Conditional Prototype Feedback (CCPF) module feeds the obtained classification probabilities back into the segmentation decoders using learned class-specific Feature-wise Linear Modulation (FWLM) parameters, which is a mechanism that is absent from cascaded and multi-head designs. Relative to previous methods, the principal advantages of CardioSynergyNet are the end-to-end differentiability of the three coupled modules, enabling joint optimization without staged pre-training; physiological grounding of the classification pathway through interpretable biomarkers, as opposed to hidden latent features; and a segmentation-refinement mechanism that iterates over the images, which is analogous to the way a cardiologist reconsiders contours after forming an initial impression. The goals of this paper are therefore four-fold:

1. To design CardioSynergyNet, a single jointly-trained network that performs cardiac segmentation, biomarker extraction, and disease classification using a shared encoder.
2. To model ED–ES cardiac motion using a CPDA module and evaluate its effect on segmentation accuracy.
3. To compute clinical biomarkers directly from soft segmentation maps using DBEL and assess their accuracy.
4. To condition segmentation on predicted disease class using a CCPF module and validate the full framework on a paired ED–ES cine-MRI cohort across five diagnostic groups.

The remainder of this paper is organized as follows: Section 2 describes the related work. Section 3 outlines the proposed model’s detailed explanation. Section 4 presents the experimental results and discussion, and Section 5 concludes the paper.

## 2. Related Work

### 2.1. Cardiac Segmentation Architectures

The U-Net encoder–decoder architecture is the most common backbone for cardiac MRI segmentation, and many efforts have been devoted to improving its components without changing the overall network topology [4]. Brahim et al. introduced ICPIU-Net, which is an architecture that extends a 3D segmentation backbone by incorporating inclusion constraints and classification priors to provide a better delineation of left ventricular myocardial disease in delayed-enhancement MRI [21]. In another study, Khened et al. demonstrated that the inclusion of papillary muscles for short-axis cine-MR segmentation can significantly improve subsequent volumetric accuracy by systematically testing the impact of anatomical choices at the preprocessing stage [22]. In a more recent study, Baccouch et al. trained and tested a series of cascaded networks to progressively localize a coarse patch to a fine patch, finding that preprocessing choices had a significant impact on the accuracy of the cascade networks in subsequent volumetric measurements [23].

Likewise, Hu et al. fused a deeply supervised network with a 3D active shape model for large-scale multi-structure segmentation, which initializes the segmentation results in an anatomically reasonable way [24,25]. More recent models, such as attention-augmented and hybrid CNN-transformer-based architectures, have further enhanced boundary delineation under the anatomical heterogeneity found in clinical cohorts. Reviews of deep architectures used for cardiac image segmentation show a definite trend toward the use of attention gating, skip-connection refinement, and self-attention blocks within otherwise convolutional pipelines [4]. In a related direction, a survey of transformer- and attention-based architectures for vascular wall segmentation shows that hybrid CNN-transformer models have demonstrated tangible improvements over well-tuned convolutional architectures across the broader field of cardiovascular imaging [26]. For LA segmentation in particular, several works have proposed encoder–decoder architectures with particular emphasis on the edges and features of the atrium, while a comprehensive survey of deep learning in atrial fibrillation imaging catalogs a continuously growing collection of shape- and boundary-aware designs for LA segmentation [9,14].

### 2.2. Joint Segmentation-Classification and Motion-Aware Multi-Task Learning

There has been a recent trend toward combining segmentation and downstream diagnostic classification within a single trainable pipeline. Early formulations used handcrafted clinical features derived from predicted segmentation maps and passed them to a separate ensemble classifier, achieving good diagnostic accuracy on the ACDC challenge but without true end-to-end differentiability between the two tasks [22]. Recent multi-task deep learning reviews [20] for medical imaging show that cascaded, parallel, and interacted designs for joint segmentation and classification are now well established across a variety of domains, including the cardiac domain. A related multi-task setup demonstrated that a fully automated multi-modal MRI network that concurrently classifies molecular subtype and segments tumors outperformed sequentially trained single-task models, lending support to the benefit of shared and cross-conditioned representations between the two tasks [15]. Similar improvements have been reported in histopathology and whole-slide imaging, where a unified multi-task network was found to perform as well as separately trained networks while requiring significantly less annotated data [27], and a related study showed that semi-supervised multi-task formulations can further reduce the number of annotated samples required for joint tasks of this type [28].

In the adult cardiac domain, multi-task learning has also been used to simultaneously segment the endocardial border and classify the severity of coronary artery disease in echocardiographic video [29], demonstrating the feasibility of the segmentation-classification coupling explored in cine-MRI and its transferability to other cardiac imaging modalities. Another line of research targets the ED-ES motion gap through the joint modeling of deformation and structure. Sinclair et al. showed that Atlas-ISTN achieves better generalization across heterogeneous cardiac datasets through joint segmentation, registration, and atlas construction in a single spatial-transformer-based network [16]. This line has been extended with biomechanics-informed priors that constrain motion field estimation to physiologically consistent motion [17], as well as with attention-guided feature fusion mechanisms that couple deformable registration with segmentation via co-attention blocks, explicitly preserving discontinuities at tissue boundaries [18]. In parallel, reconstruction-side work suggests the use of learned, differentiable operators in place of classical iterative methods once sufficient training data are available: a recent study on deep separable spatiotemporal networks for dynamic cardiac MRI highlights the benefits of differentiable, learnable blocks within segmentation-classification pipelines over hard, non-differentiable post-processing [30].

### 2.3. Biomarker-Driven and Feedback-Conditioned Coupling

A smaller but methodologically significant body of work aims to make the relationship between segmentation and classification explicit through clinically interpretable intermediate representations rather than relying solely on shared latent features. AtrialJSQnet directly incorporates spatial and shape priors into a joint segmentation-quantification objective for the left atrium, and it has been shown to improve anatomical and quantitative accuracy by supervising segmentation on a set of clinical quantities in addition to pixel-level labels [12]. Similar results have been reported for non-contrast computed tomography (CT) of left atrial appendage (LAA) volumetry, which has been directly compared against clinically important endpoints [14]. Multi-stage designs that pass information between preliminary segmentation and subsequent classification stages have been found to improve the consistency of diagnostic judgments across stages [31]. In addition, reviews of multi-task architectures report that designs which explicitly re-inject the intermediate predictions of one task to condition the other, known as interacted designs, consistently outperform designs relying only on a shared encoder, across the medical imaging application domains surveyed [20]. AI-enhanced ECGs [32] serve as scalable digital biomarkers, leveraging deep learning on standard and wearable tracings to detect rhythm disorders, structural heart diseases, acute coronary events, and long-term risk. However, widespread clinical adoption still requires prospective multicenter validation, improved model interpretability, and seamless workflow integration.

## 3. Methodology

### 3.1. Overall Architecture and System Workflow

**Figure 1:**
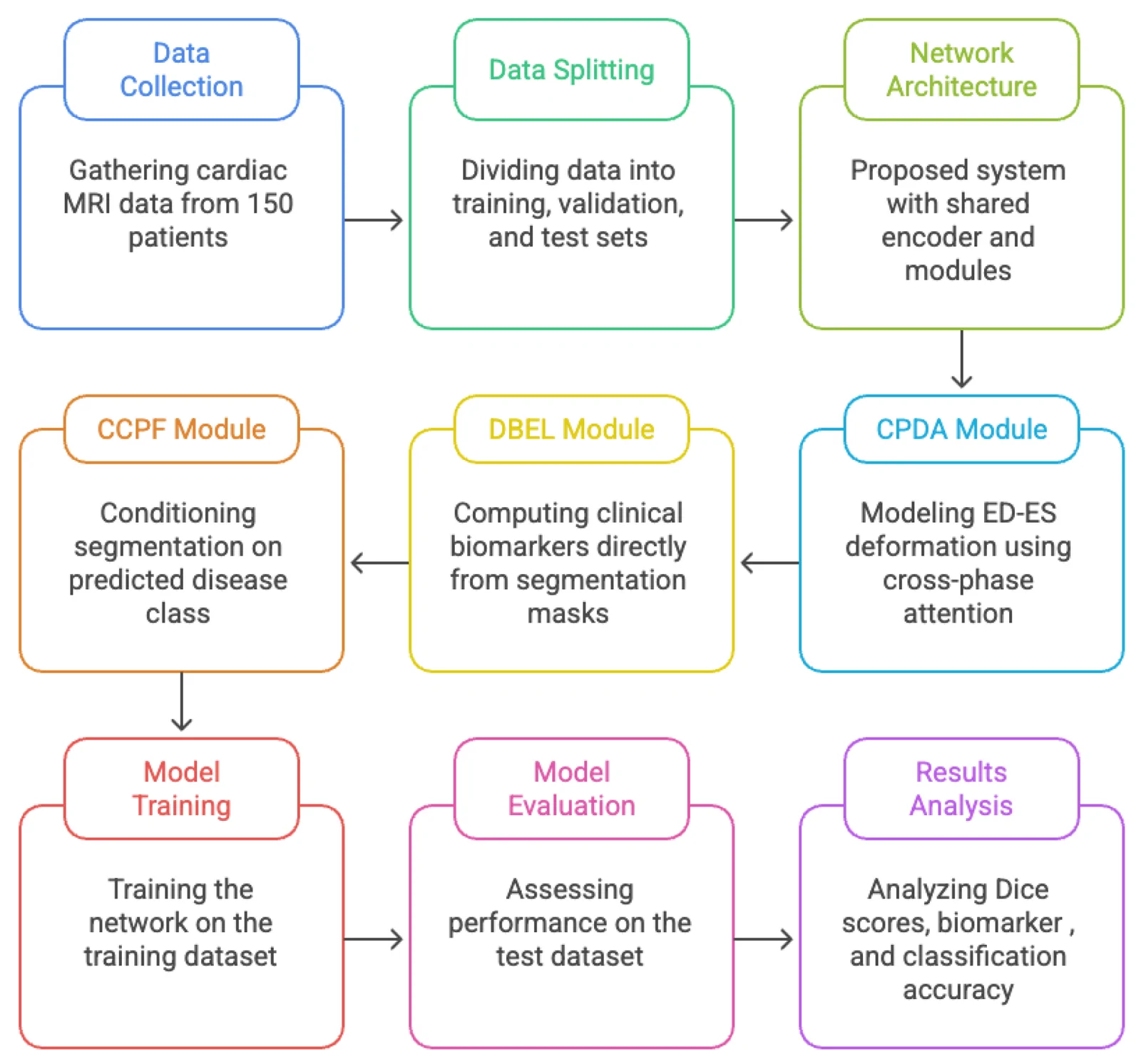
Overview of the CardioSynergyNet workflow, including data preparation, the proposed network architecture (CPDA, DBEL, and CCPF modules), model training, and evaluation.

CardioSynergyNet jointly performs cardiac segmentation, biomarker estimation, and disease classification within a single trained network, using paired end-diastolic (ED) and end-systolic (ES) cine-MRI slices processed through a weight-shared encoder. Three modules link the tasks together: Cross-Phase Deformation Attention (CPDA) aligns ED and ES features by modeling the deformation between phases, a Differentiable Biomarker Extraction Layer (DBEL) computes eight clinical biomarkers directly from the resulting soft segmentation masks, and Class-Conditional Prototype Feedback (CCPF) uses the predicted disease class to refine the segmentation in a second decoding pass. This creates a closed loop rather than a one-way pipeline—segmentation informs the biomarkers and diagnosis, and the diagnosis feeds back to sharpen the segmentation. The model was trained end-to-end on 150 patients across five diagnostic groups, and the individual modules, along with the training procedure, are detailed in the following sections. The overall architecture and workflow of the proposed CardioSynergyNet framework is illustrated in Figure 1.

### 3.2. Data Pipeline and Preprocessing Module

**Figure 2:**
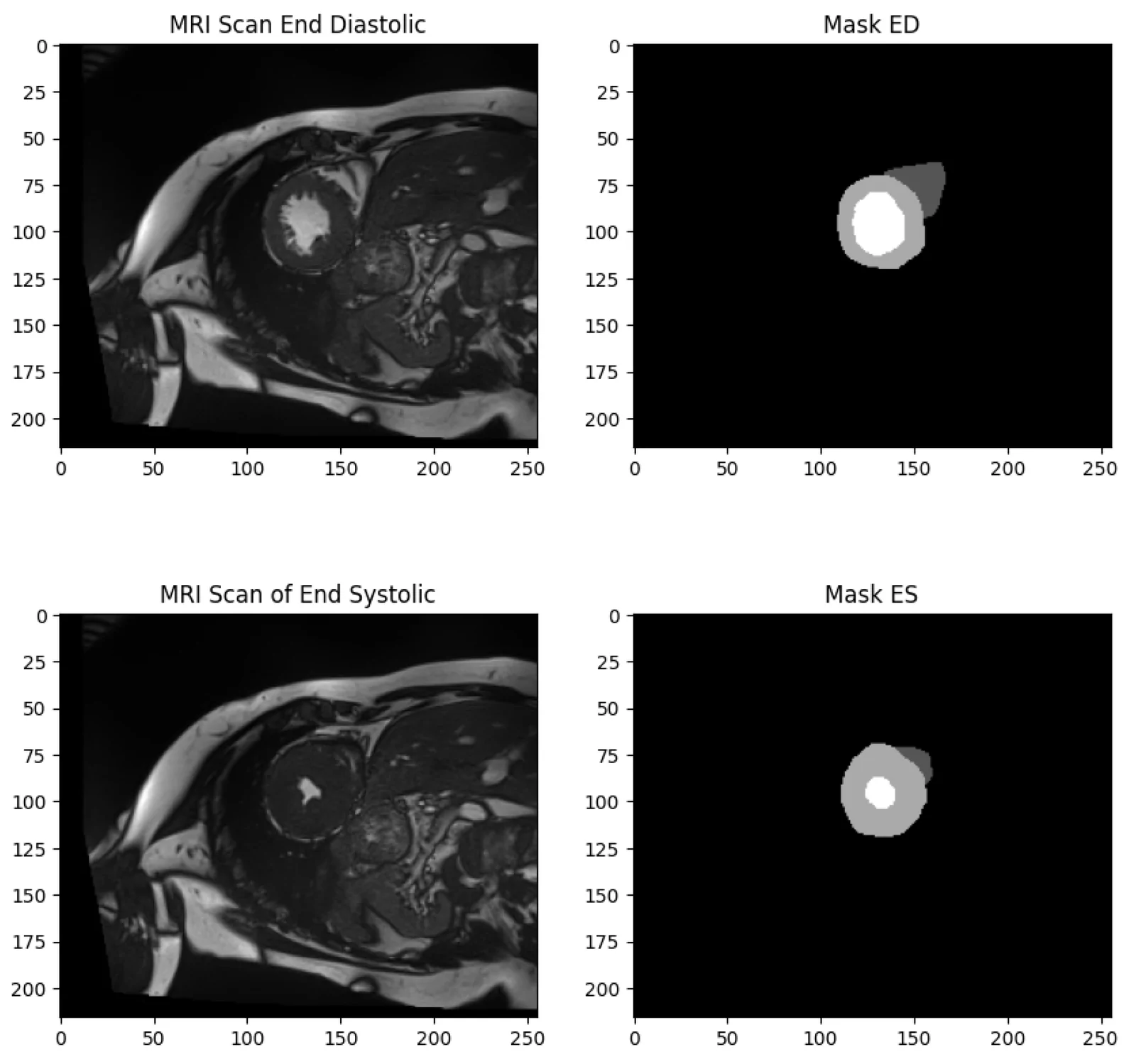
Sample cardiac MRI slice and corresponding segmentation mask at end-diastole (ED) and end-systole (ES).

The data pipeline module forms the foundational layer of the CardioSynergyNet framework, which is responsible for ingesting, organizing, and preparing cardiac cine-MRI data from the Automated Cardiac Diagnosis Challenge (ACDC) dataset for downstream model training. It encompasses patient-level metadata discovery, frame-pair extraction, voxel-level preprocessing, and stratified data splitting. Each patient’s directory is parsed to extract end-diastolic (ED) and end-systolic (ES) frame indices from their corresponding configuration files, which are then matched to their respective NIfTI volume files. Ground-truth segmentation masks—covering the right ventricle (RV), myocardium (Myo), and left ventricle (LV)—are loaded alongside the image volumes, and per-patient clinical attributes such as height, weight, and number of cine frames are retained throughout. A representative data sample, showing the MRI scan and corresponding segmentation mask at both end-diastolic (ED) and end-systolic (ES) phases, is illustrated in Figure 2.

Preprocessing operates slice-by-slice on the 3D volumes. Each 2D slice is first resampled to a uniform isotropic spacing of 1.4 mm × 1.4 mm using bilinear interpolation for images and nearest-neighbor interpolation for masks, preserving label integrity. The resampled slices are then center-cropped or zero-padded to a fixed spatial resolution of 224 × 224 pixels. Intensity normalization is applied per-volume using the 5th–95th percentile range, clipping extreme values before linearly rescaling to [0, 1]. Paired ED-ES slice correspondences are built by matching each ED slice index to its proportionally equivalent ES slice via relative depth. To prevent patient-level data leakage, the dataset is partitioned at the patient level using a stratified scheme that preserves class distribution across train, validation, and test splits. A module-level LRU cache is employed to avoid redundant disk I/O across DataLoader workers, substantially reducing preprocessing overhead during multi-epoch training.

Adopting a unified preprocessing pipeline across all splits ensures strict consistency between training and evaluation conditions. The stratified patient-level split eliminates the risk of slice-level leakage that would artificially inflate performance metrics. Furthermore, caching preprocessed volumes at the worker level reduces I/O bottlenecks significantly on limited-memory systems.

### 3.3. Cross-Phase Deformation Attention (CPDA)

The Cross-Phase Deformation Attention (CPDA) module is a spatially adaptive feature alignment mechanism designed to register and fuse bottleneck-level feature representations extracted from the two cardiac phases—end-diastole and end-systole. Rather than simply averaging or concatenating phase features, CPDA explicitly estimates a deformation flow field that captures the spatial displacement between the two phases, and it uses a learned confidence map to selectively weight the warped ED features against the original ES features during fusion. This design reflects the physiological reality that the heart undergoes significant shape change between ED and ES and that naive feature averaging discards this motion signal entirely.

**Figure 3:**
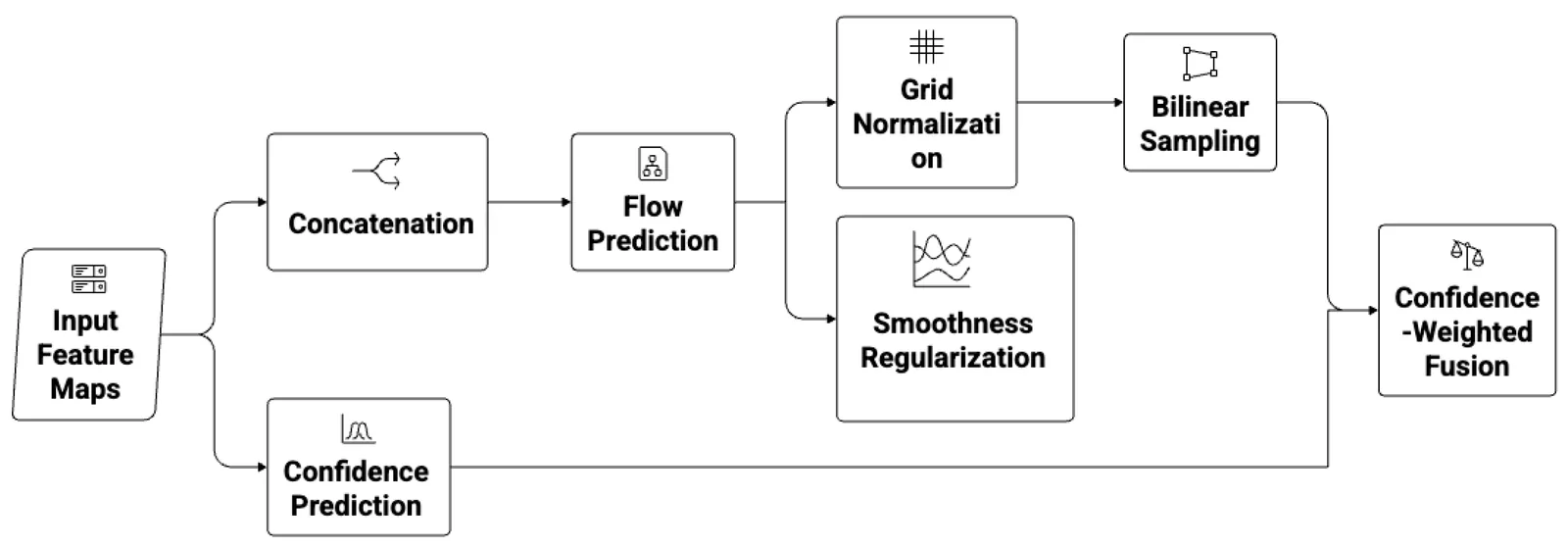
Architecture of the Cross-Phase Deformation Attention module.

Concretely, the module receives a pair of bottleneck feature maps, each of shape (B, C, H, W), which are extracted from the shared-weight DualPhaseEncoder. These maps are concatenated along the channel dimension and passed through a lightweight three-layer convolutional flow predictor, which outputs a two-channel displacement field. This field is normalized to the [−1, 1] grid coordinate system and added to an identity sampling grid. The resulting deformed grid is applied to the ED feature map via differentiable bilinear grid sampling, producing a spatially warped version, as shown in Figure 3. A separate confidence predictor—also convolutional—produces a sigmoid-gated scalar map indicating where the ES features should be trusted over the warped ED features. The final fused representation is a confidence-weighted blend of ES features and warped ED features. A smoothness regularization penalty, computed as the mean absolute spatial gradient of the flow field, is applied during training to prevent degenerate deformations.

CPDA enables the model to explicitly account for cardiac motion between phases rather than treating ED and ES as independent or exchangeable inputs. The differentiable warping ensures that gradient signals flow back through the spatial transformation, allowing flow estimation to be jointly optimized with segmentation and classification objectives. Confidence-gated fusion further prevents noisy deformation estimates from corrupting the representation in early training.

### 3.4. Differentiable Biomarker Extraction Layer (DBEL)

The Differentiable Biomarker Extraction Layer (DBEL) is a fully differentiable, anatomy-aware module that computes an 8-dimensional vector of clinically meaningful cardiac biomarkers directly from the soft segmentation probability maps produced by the model’s decoders—without requiring hard argmax operations at any point. The biomarkers include a left ventricular ejection fraction proxy (derived from the fractional change in LV area between ED and ES), myocardial mass proxy, RV-to-LV area ratio at ED, myocardial wall thickness at ED, and individual RV and LV region areas at both cardiac phases. All quantities are expressed in physical units using the known voxel spacing (mm^2^), making them interpretable as physiological measurements rather than arbitrary latent values.

**Figure 4:**
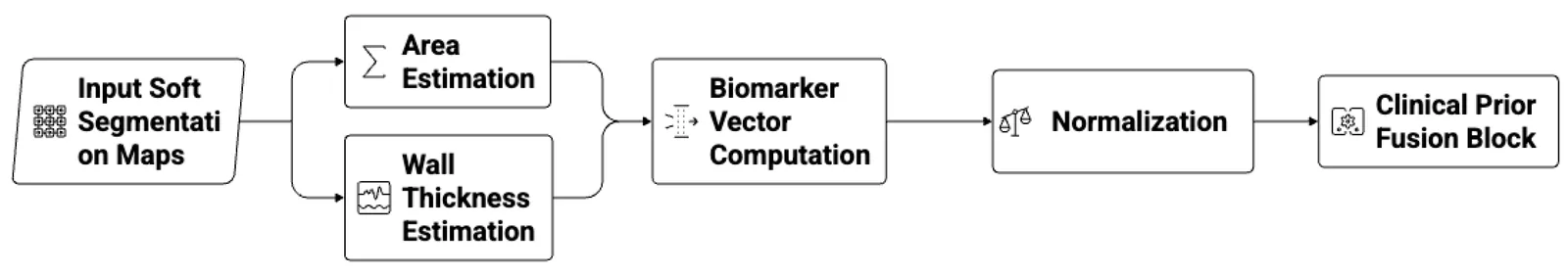
Overview of the Differentiable Biomarker Extraction Layer.

The DBEL receives soft segmentation maps—computed via softmax over the logits from both the ED and ES decoders—as input. Area estimates for each cardiac structure are computed by summing the corresponding soft probability channel over all spatial positions and multiplying by the voxel area in mm^2^. Wall thickness is estimated through a differentiable soft erosion operation: the myocardium probability channel is eroded by applying a max-pooling operation with a negated input, and the resulting erosion residual (i.e., the soft ring) approximates boundary pixel density, as depicted in Figure 4. Dividing by the square root of voxel area converts this to a thickness estimate in millimeters. All operations are implemented using standard PyTorch (v2.10.0) primitives (sum, clamp, F.max_pool2d), meaning gradients propagate freely through every biomarker computation back to the segmentation logits. The resulting biomarker vector is normalized by fixed anatomical reference scales and fed into the Clinical Prior Fusion Block for classification conditioning.

By bridging segmentation outputs and classification inputs through explicit physiological quantities, DBEL forces the segmentation decoders to produce anatomically consistent masks—not just pixel-accurate ones. Gradients from the classification loss flow back through the biomarkers into the decoders, incentivizing them to preserve clinically relevant structural relationships. The fully differentiable design avoids the vanishing-gradient problem that would arise if hard thresholding were used.

### 3.5. Class-Conditional Prototype Feedback (CCPF)

The Class-Conditional Prototype Feedback (CCPF) module is a learned feedback mechanism that closes the loop between classification predictions and pixel-level segmentation outputs by conditioning the segmentation decoders on the model’s own diagnostic prediction. For each of the five cardiac pathology classes—Normal, Myocardial Infarction, Dilated Cardiomyopathy, Hypertrophic Cardiomyopathy, and Abnormal Right Ventricle—a trainable prototype embedding is maintained in a learnable parameter matrix. At inference, the model’s class probability distribution is used to compute a soft, weighted combination of these prototypes, which is then transformed into a pair of Feature-wise Linear Modulation (FWLM) parameters: a channel-wise scale (gamma) and shift (beta). These parameters are injected into the final feature map of each segmentation decoder, directly modulating spatial responses based on the predicted pathology.

**Figure 5:**
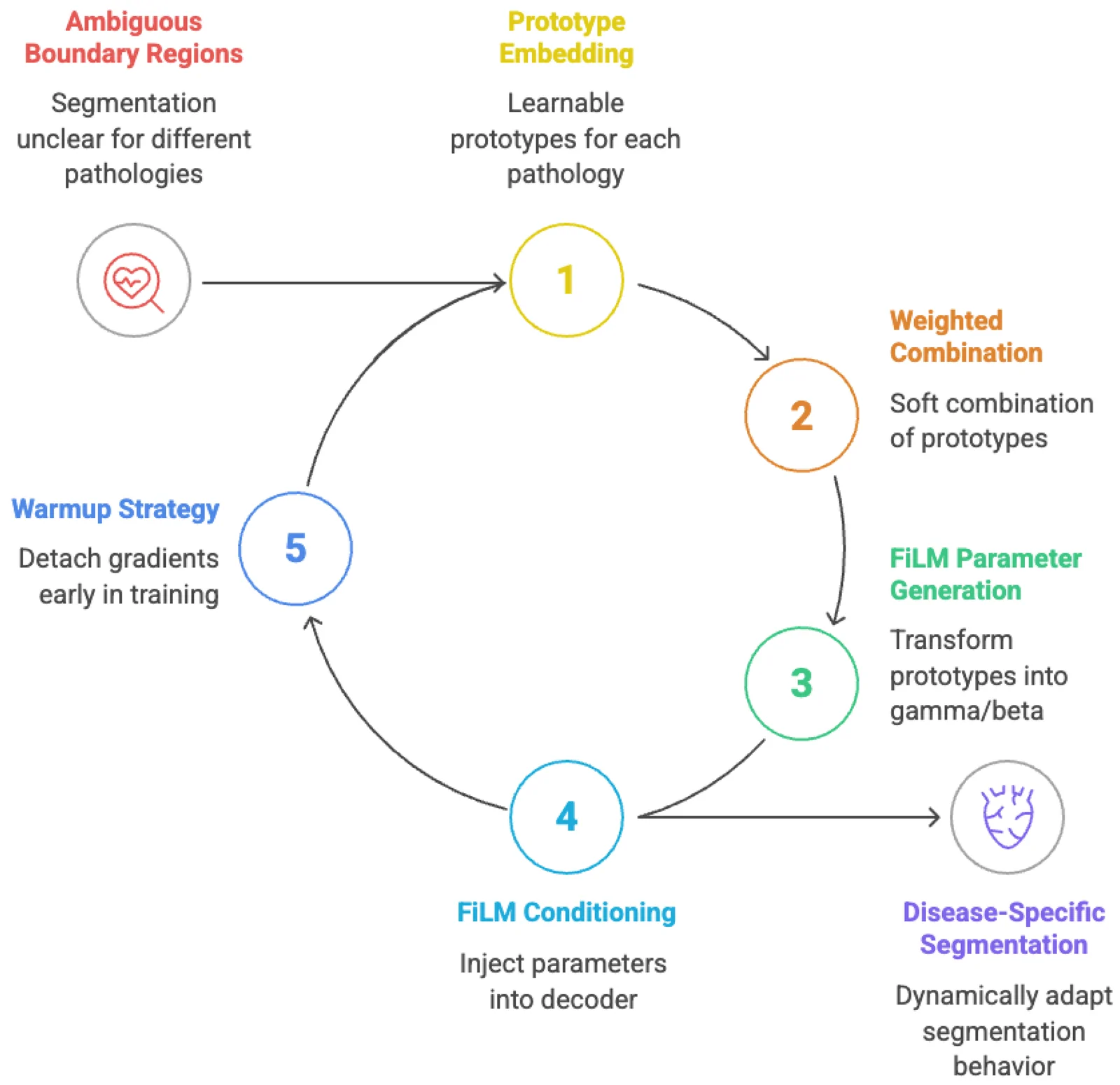
Overview of the Class-Conditional Prototype Feedback module.

The CCPF operates in two decoder passes. In the first pass, segmentation logits are produced without any FiLM conditioning; these preliminary logits are used by the DBEL to compute soft biomarkers and subsequently drive the classification head. In the second pass, the resulting class probabilities are fed into the CCPF module, which produces gamma and beta tensors broadcastable across the spatial dimensions of the decoder’s penultimate feature map, as shown in Figure 5. The FiLM operation scales and shifts this feature map before the final 1 × 1 classification convolution produces segmentation logits. During early training, the class probability inputs to the CCPF are detached from the computational graph—a warmup strategy that prevents noisy early-epoch classifications from corrupting decoder gradients before the backbone has learned basic anatomical representations. After the warmup period, the full gradient path is restored.

CCPF allows the model to dynamically adapt its segmentation behavior based on the predicted pathology class, effectively implementing a soft, learned form of disease-specific segmentation. This bidirectional coupling between classification and segmentation is a key architectural distinction that enables the model to resolve ambiguous boundary regions differently for, say, hypertrophic versus dilated cardiomyopathy. The FiLM-based conditioning is lightweight—adding only two linear layers per class—yet has been shown in prior work to be a highly expressive modulation mechanism for conditional prediction.

### 3.6. Joint Loss Function and Training Schedule

Training CardioSynergyNet involves optimizing a multi-task composite loss that jointly supervises segmentation, classification, biomarker regression, and deformation smoothness. The segmentation loss is a weighted combination of Dice loss and class-weighted cross-entropy applied independently to both the ED and ES decoder outputs. The Dice loss is computed per-sample over spatial dimensions only; then, it is averaged across foreground classes and the batch—an important correction over the naive formulation that averages across batch and spatial jointly, which suppresses the gradient signal from rare foreground structures in small batches. Inverse-frequency class weights for the cross-entropy component are set to reflect the approximate background-to-foreground pixel ratio in ACDC, preventing the model from collapsing to predicting background everywhere. The classification objective is a standard softmax cross-entropy loss. Biomarker regression is supervised using Smooth L1 loss against ground-truth biomarker values computed from the hard segmentation masks. A smoothness penalty on the CPDA flow field is added to prevent degenerate spatial deformations.

The biomarker loss weight is ramped linearly from zero to its maximum value over the first 15 training epochs. This curriculum is motivated by the observation that DBEL inputs—soft segmentation maps—are near-uniform probability distributions at the start of training, producing biomarker estimates that are essentially noise. Applying a large biomarker gradient during this phase would corrupt the segmentation decoders before they have learned basic structural anatomy. Optimization is performed using AdamW with a weight decay of 1 × 10^−4^. The learning rate follows a sequential schedule: a 5-epoch linear warmup from 10% of the peak rate, which is followed by cosine annealing to 1% of the peak over the remaining epochs. Model selection is performed on a composite validation score combining mean ED + ES Dice and classification accuracy with a patience of 15 epochs for early stopping.

The curriculum on biomarker loss stabilizes early training and prevents the multi-task objective from pulling the model in conflicting gradient directions during the initial epochs. Combined with LR warmup, this schedule reduces the risk of early gradient explosion associated with large models trained on small medical imaging datasets. Selecting the best checkpoint on a combined metric ensures that neither segmentation nor classification performance is sacrificed for the other.

## 4. Results

CardioSynergyNet was trained and evaluated on 150 patients drawn in equal number from five diagnostic groups (Normal, Myocardial Infarction, Dilated Cardiomyopathy, Hypertrophic Cardiomyopathy, and Abnormal Right Ventricle), giving 1489 paired end-diastolic (ED)/end-systolic (ES) slices in total. The data were split at the patient level into 110 training, 20 validation, and 20 test patients (1103/189/197 slices, respectively) so that no patient appears in more than one split. The final model has 77,516,816 trainable parameters and was trained for 80 epochs with the checkpoint from epoch 78 kept for evaluation because it gave the best validation Dice score and validation classification accuracy. All numbers reported below come from the held-out test set unless stated otherwise.

### 4.1. Training Behavior

**Figure 6:**
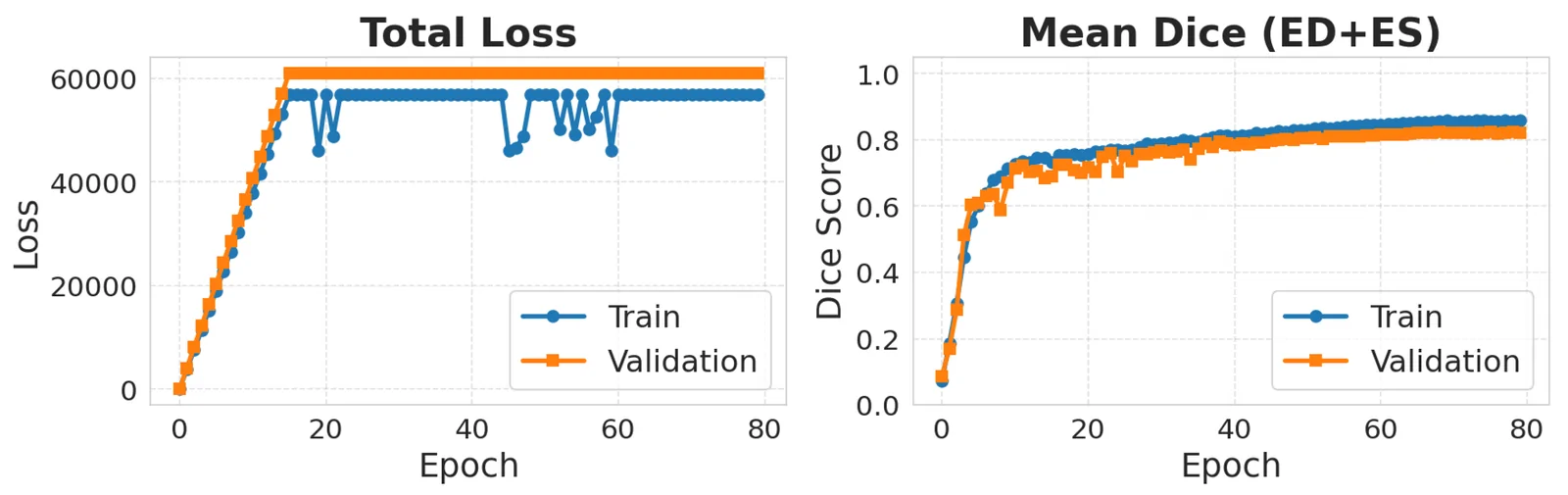
Training and validation curves for CardioSynergyNet over 80 epochs. (**Left**): total loss. (**Right**): mean Dice score averaged over the ED and ES segmentation outputs. The validation curve tracks the training curve closely, indicating that the model generalizes reasonably well rather than memorising the training set.

Figure 6 plots the total loss and the mean Dice score against epoch number for both the training set and the validation set. The loss curve rises sharply during the first 10–15 epochs before flattening out and is mainly caused by the biomarker regression term, whose scale is large compared with the segmentation and classification losses, dominating the total loss as soon as it switches on. After that, point training and validation loss move almost together with a few short dips around epochs 20, 45, 50, and 58 that line up with drops in the learning-rate schedule rather than with overfitting. The Dice curve tells a cleaner story. Both curves climb quickly in the first 10 epochs, reaching roughly 0.7, and then continue to improve slowly until they plateau close to epoch 60. Training and validation Dice stay close to one another throughout with a gap of around 0.02–0.03 Dice points by the end of training, which suggests the segmentation branch is not overfitting badly despite the size of the network.

### 4.2. Overall Segmentation Performance

On the test set, the model reaches a mean Dice score of 0.9050 (computed across all four classes, including background) with the ED phase clearly outperforming the ES phase (0.8430). A paired *t*-test was run between the per-patient ED and ES Dice scores to check whether this gap is statistically meaningful, and it was: t=5.88, p<0.0001.

Breaking the Dice score down by class (Table 1) confirms this. Background segmentation is almost perfect in both phases (Dice >0.997), which is unsurprising since background pixels dominate the image. The left ventricle (LV) is segmented well in both phases (0.9266 at ED, 0.8522 at ES), and the myocardium (Myo) is fairly stable across phases (0.8437 at ED versus 0.8291 at ES). The right ventricle (RV), however, drops sharply from 0.8542 at ED to 0.6931 at ES—a fall of over 16 Dice points. The RV is the structure that changes shape the most between the two phases and has the thinnest, most irregular wall, so this drop fits the expected difficulty pattern rather than pointing to a specific flaw in the model.

### 4.3. Segmentation Performance Across Disease Groups

**Figure 7:**
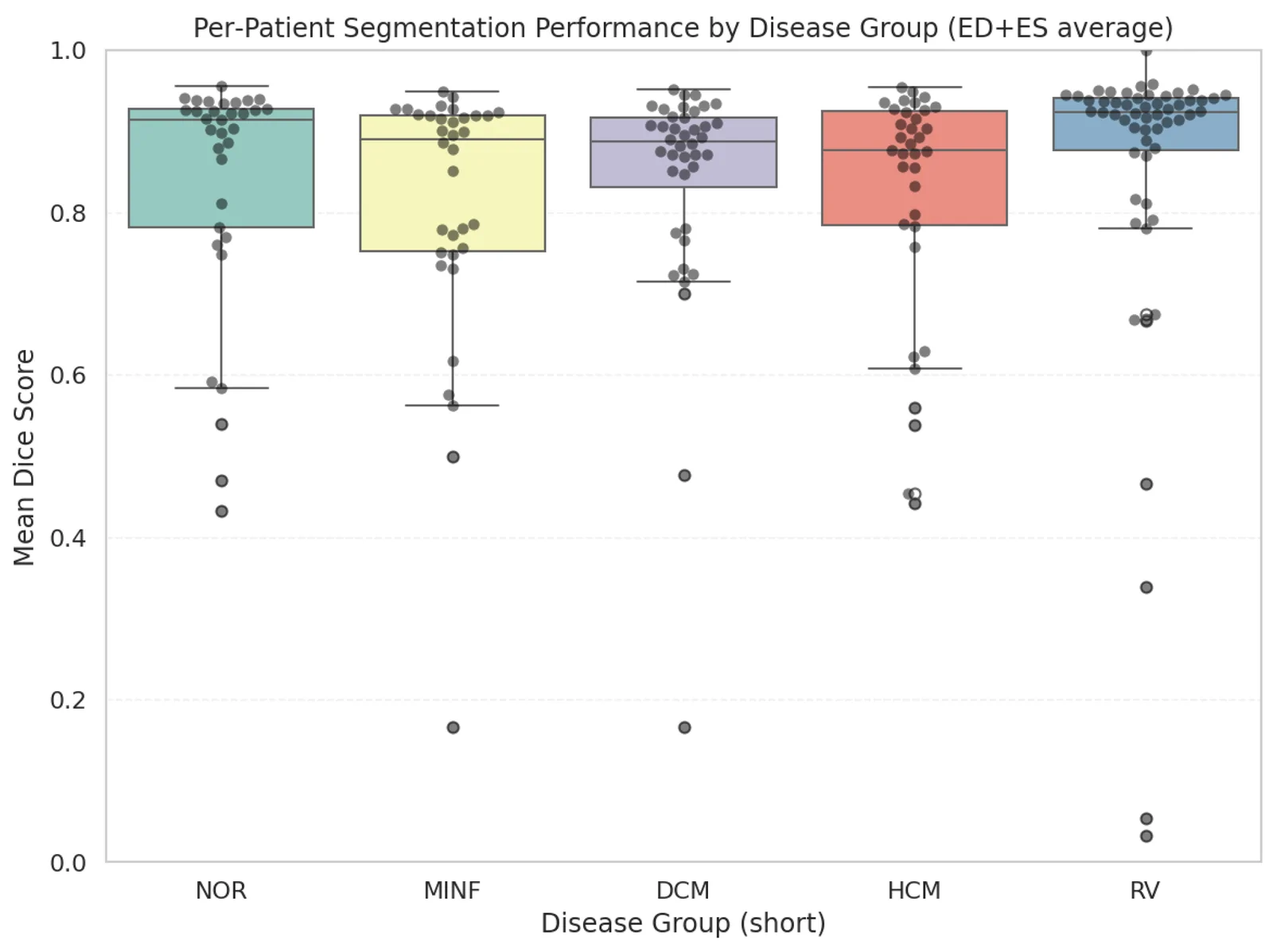
Slice-level Dice score distribution per disease group on the test set (ED and ES phases combined). Each dot is one slice; the box marks the interquartile range. Low-Dice outliers correspond mainly to apical/basal slices where the cardiac structures are small.

Figure 7 shows the spread of slice-level Dice scores for each of the five disease groups with individual points jittered on top of the boxes so the underlying distribution is visible rather than hidden behind a summary statistic. Median performance is broadly similar across groups, sitting between 0.85 and 0.92 Dice, which suggests the model has not specialized on one disease at the expense of the others. The Abnormal Right Ventricle (RV) group, the Myocardial Infarction (MINF) group, and the Dilated Cardiomyopathy (DCM) group all show a handful of slices where Dice falls below 0.2, pulling the lower whisker down a long way.

This matches the per-disease, per-phase breakdown in Table 2: every disease class loses Dice when moving from ED to ES, but the size of that drop varies. Hypertrophic Cardiomyopathy showed the largest drop (0.8910 to 0.7493). While myocardial thickening may hinder end-systolic RV boundary delineation, treating this descriptive explanation as a mechanism requires validation (e.g., correlating wall thickness with RV error).

### 4.4. Qualitative Segmentation Results

**Figure 8:**
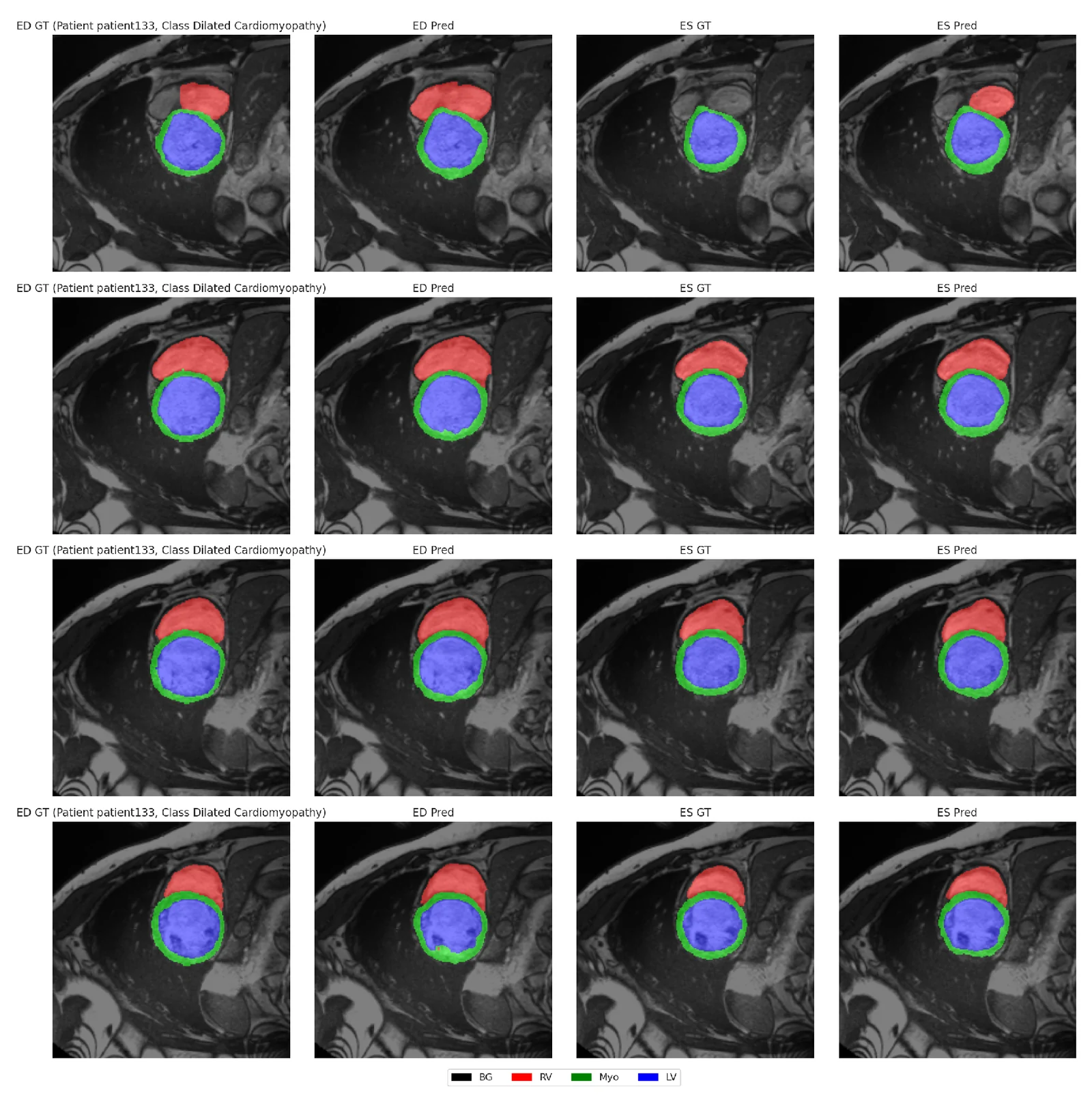
Qualitative comparison between ground-truth and predicted segmentation masks for four slices of a Dilated Cardiomyopathy test patient. Columns, left to right: ED ground truth, ED prediction, ES ground truth, ES prediction. Color code: red = right ventricle, green = myocardium, blue = left ventricle, black = background.

To complement the quantitative scores, Figure 8 shows predicted segmentation masks next to the ground truth for four consecutive slices from a single test patient (a Dilated Cardiomyopathy case) at both ED and ES. Visually, the predicted masks line up closely with the ground truth: the RV (red), myocardium (green), and LV (blue) boundaries are smooth and the relative positions of the three structures are preserved across all four slices. Minor disagreement is visible mainly at the RV–myocardium boundary, where the predicted RV region is slightly smaller than the ground-truth RV in some slices consistent with the lower RV Dice score reported in Table 1. Additional qualitative examples, including both successful and failure cases, are provided in the Supplementary Materials (Figures S1–S6).

### 4.5. Cross-Phase Deformation Attention

**Figure 9:**
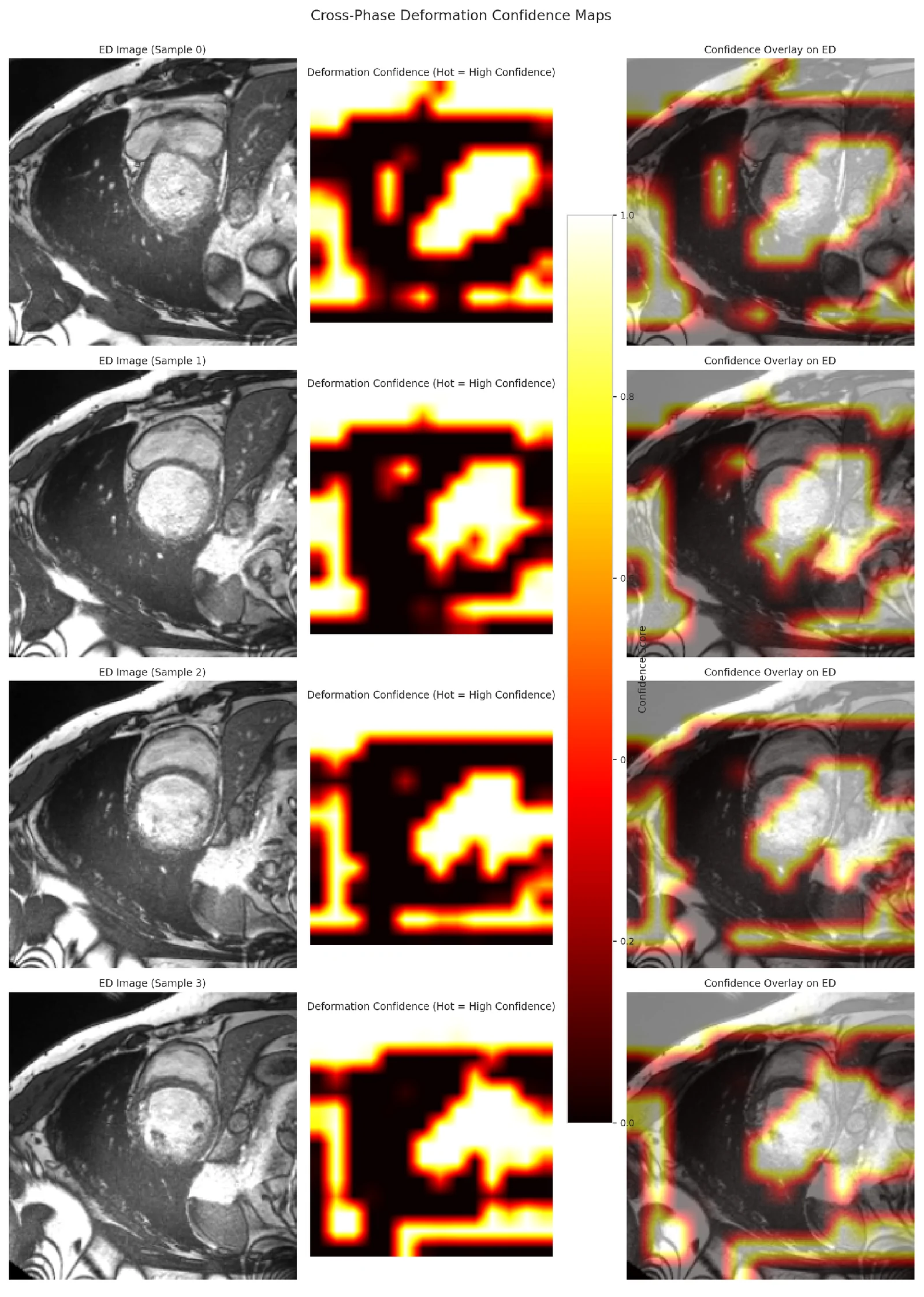
Cross-Phase Deformation Attention confidence maps for four representative ED slices. (**Left**): original ED image. (**Middle**): predicted confidence map (brighter = higher confidence). (**Right**): confidence map overlaid on the ED image. High confidence concentrates around the myocardial boundary, which is the region most affected by ED-to-ES deformation.

The Cross-Phase Deformation Attention (CPDA) module produces, alongside the predicted deformation flow, a confidence map that indicates how much the network trusts its own ED-to-ES alignment at each spatial location. Figure 9 shows this confidence map for four ED images, which is overlaid on the original scan. The confidence is consistently highest around the boundary of the left ventricle and the surrounding myocardial ring, shown as the bright yellow/white outline, while the background and the chamber interior receive low confidence. The boundary region is where ED and ES anatomy differ most due to contraction, so it is plausibly the region where a deformation estimate carries the most diagnostic information; the concentration of high confidence there is consistent with, but does not by itself confirm, the model having learned to prioritize this region for that reason.

### 4.6. Biomarker Estimation

**Figure 10:**
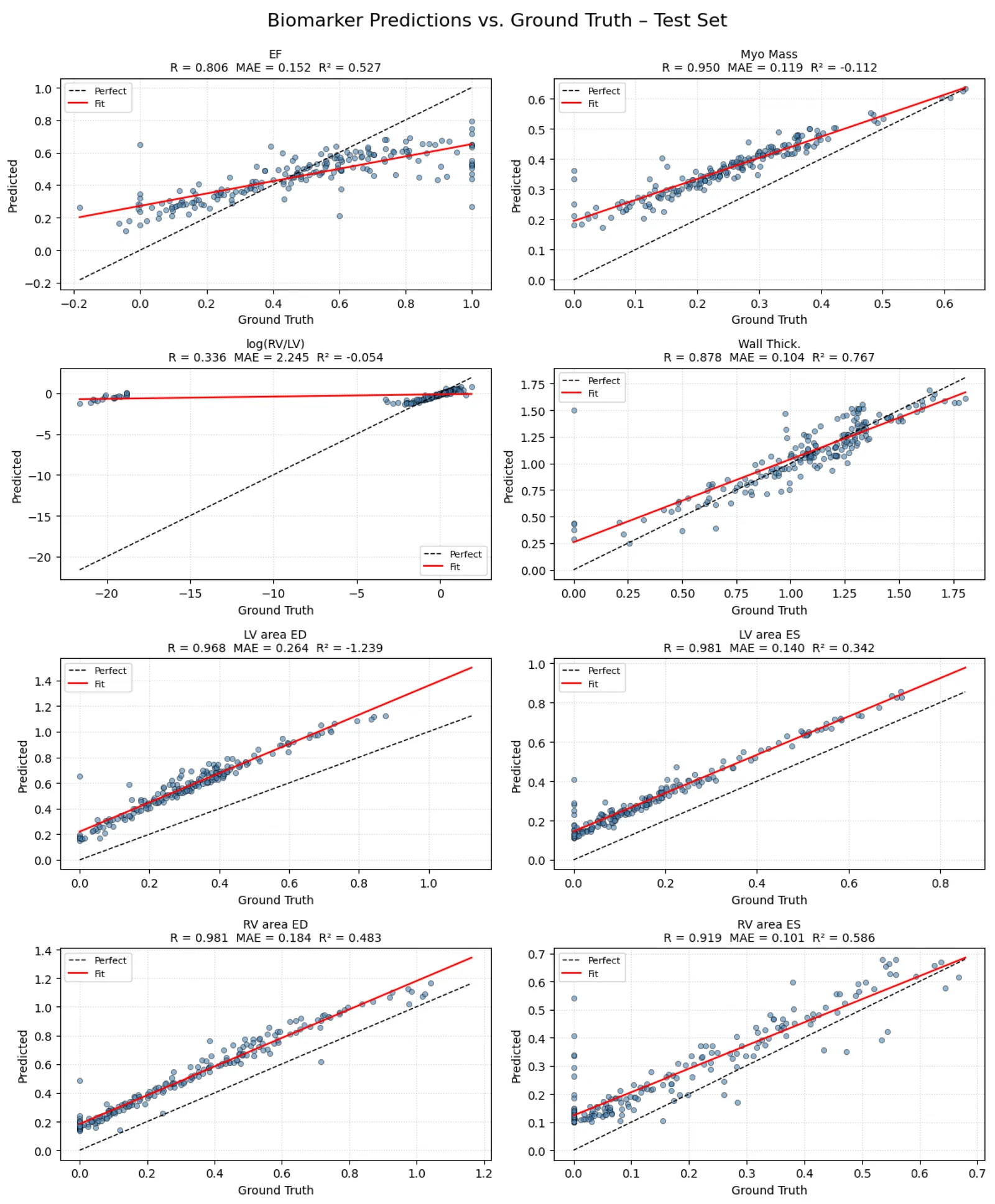
Predicted versus ground-truth values for all eight biomarkers on the test set. The dashed line marks perfect agreement, while the red line is the linear fit. Most biomarkers cluster reasonably close to the identity line; log(RV/LV) (second row, left) shows the model collapsing toward a near-constant prediction rather than tracking the true spread of the ratio.

The DBEL reads eight clinical biomarkers directly off the soft segmentation masks so that they can be supervised end to end. Table 3 reports the mean absolute error (MAE) and Pearson correlation (R) between predicted and ground-truth values for each biomarker on the test set; Figure 10 shows the corresponding scatter plots.

Six of the eight biomarkers correlate well with the ground truth. The four chamber-area biomarkers are the strongest performers with R ranging from 0.919 (RV area, ES) up to 0.981 (LV area, ES); myocardial mass proxy follows closely at R = 0.950, and wall thickness reaches R = 0.878. Looking at the corresponding panels in Figure 10, the points sit close to the fitted line and track the identity line reasonably well at higher ground-truth values. A consistent pattern does emerge at the low end, though: predictions tend to sit above the identity line for small ground-truth areas, so the model is mildly over-predicting when the true chamber or wall is small. This is visible across all four area biomarkers and wall thickness alike, and it is worth flagging even where R is high, since R rewards rank ordering rather than calibration at the extremes.

EF proxy trails the well-behaved group at R = 0.806 and MAE = 0.152. Its scatter plot (top-left panel) tells the story better than the number does: predictions crowd into a narrow band roughly between 0.2 and 0.7, while the ground truth stretches from about −0.2 to 1.0. The fitted line is noticeably flatter than the identity line, particularly past a ground truth of 0.6, where predictions plateau instead of climbing further. So, the model captures the overall trend in EF but compresses the extremes, which is a fairly typical failure mode for a proxy biomarker derived from area ratios rather than measured directly.

log(RV/LV) is the clear outlier of the set. R drops to 0.336 and MAE balloons to 2.245, which are both far outside the range of the other six biomarkers. The scatter plot makes the reason obvious: almost every prediction places in a tight band between about −1 and 0 regardless of what the ground truth is doing, and the ground truth itself ranges from roughly −22 to 2. The fitted line is nearly flat, which is the signature of a model that has learned the typical value of the ratio but not its patient-to-patient variation. Because the log transform was meant to tame the instability of a raw area ratio (RV and LV areas both approach zero on basal and apical slices), this suggests the log-ratio target is still difficult for the network to learn directly rather than that the underlying areas are being estimated badly—LV and RV areas are, after all, the best-predicted biomarkers in the table.

### 4.7. Area Agreement at the Slice Level

**Figure 11:**
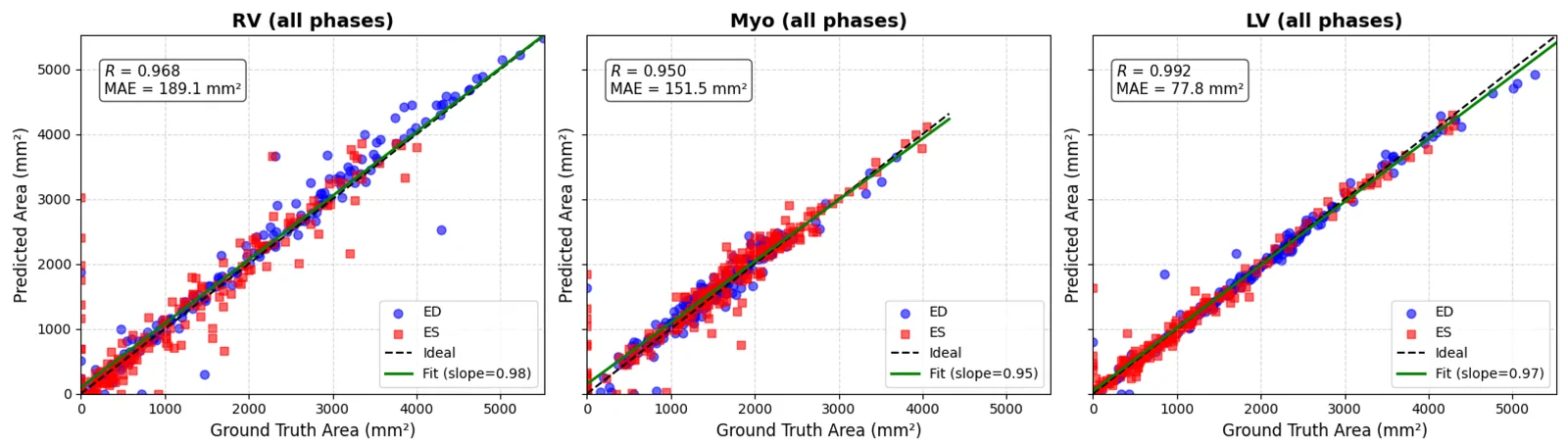
Predicted versus ground-truth structure area (mm^2^) for RV, myocardium, and LV, which are pooled over ED and ES phases across the test set. The dashed black line is the line of perfect agreement; the solid green line is the linear fit to the predicted points.

Biomarkers are themselves derived from predicted areas, so it is useful to look at area agreement directly independent of any biomarker normalization. Figure 11 plots predicted versus ground-truth area, in mm^2^, for the RV, myocardium, and LV with ED and ES slices shown separately and pooled across the whole test set. All three structures show a strong linear relationship with the identity line: LV area has the tightest fit (R = 0.992, MAE = 77.8 mm^2^), which is followed by the myocardium (R = 0.950, MAE = 151.5 mm^2^) and the RV (R = 0.968, MAE = 189.1 mm^2^). The fitted regression lines (green) sit almost on top of the ideal line (black dashed) for all three structures, indicating only a small systematic bias once outliers are accounted for. The RV plot shows the most scatter of the three with a handful of points where the predicted area diverges noticeably from ground truth at low-to-mid area values; this lines up with the lower RV Dice score reported earlier and reflects the same underlying difficulty in delineating a thin, variably shaped structure.

### 4.8. Disease Classification Performance

**Figure 12:**
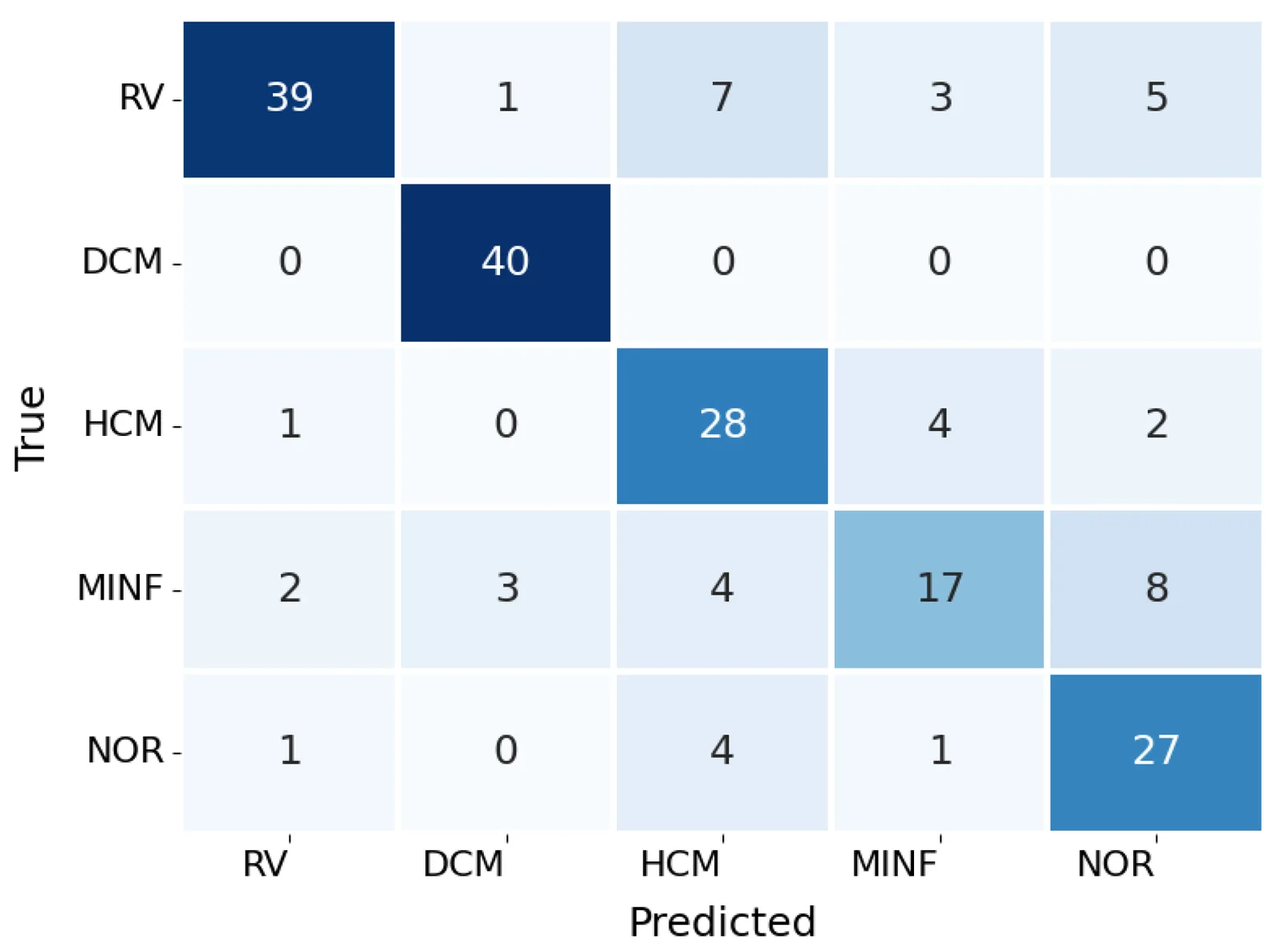
Confusion matrix for the five-class disease classification task on the test set (slice-level; N = 197 test slices from 20 patients). RV = Abnormal Right Ventricle, DCM = Dilated Cardiomyopathy, HCM = Hypertrophic Cardiomyopathy, MINF = Myocardial Infarction, NOR = Normal.

The classification head reaches an overall test accuracy of 76.65% with a macro-averaged F1-score of 0.7525 across the five distinct classes. Figure 12 shows the confusion matrix with rows giving the true class and columns the predicted class. Unlike the segmentation metrics reported above, which are aggregated per patient, classification accuracy and the confusion matrix are computed at the slice level (197 test slices from 20 patients).

RV is classified correctly in 39 of 55 cases with the main confusion being toward HCM (7 cases) and a smaller number toward DCM (1), MINF (3), and NOR (5). DCM is the cleanest class, with 40 of 40 cases correctly classified and zero confusion with any other disease, which fits with DCM’s distinctive radiological signature of an enlarged, thin-walled LV. HCM is correctly identified in 28 of 35 cases, with the remaining cases spread fairly across the other four classes, suggesting no single confusable disease but rather a generally harder class. MINF is the weakest class in this matrix with only 17 of 34 cases correctly identified; it is most often confused with NOR (8 cases), which is plausible since both conditions can present with regional wall-motion or shape abnormalities that overlap in appearance on a single static frame. NOR itself is recognized in 27 of 33 cases with the remaining cases split between RV, HCM, and MINF.

### 4.9. Summary

Across the three tasks, the model performs most reliably on segmentation, reaching a mean test Dice of 0.9050 (computed across all four classes, including background). DBEL significantly reduced segmentation Dice (p<0.001), showing that differentiable biomarker computation comes at the cost of segmentation accuracy. I accept this trade-off because it enables otherwise impossible biomarker estimation—not because it boosts overall model performance. Biomarker regression works well for area-based and mass-based quantities (R typically above 0.85) but degrades for ratio-based quantities such as EF proxy and especially RV/LV ratio, both of which inherit error from dividing by small predicted areas. Disease classification reaches a respectable 76.65% accuracy with confusions occurring mainly between diseases that are genuinely difficult to separate from imaging appearance alone. These results, taken together, support the central design idea of CardioSynergyNet: that segmentation, biomarker extraction, and classification can be trained jointly and still reach competitive performance on each individual task, while the qualitative segmentation examples and CPDA confidence maps (Figure 8 and Figure 9) are visually consistent with the model attending to anatomically plausible regions, such as the myocardial boundary. These visualizations are illustrative on a small number of cases rather than a systematic or quantitative verification of the model’s decision basis, and they should not be interpreted as ruling out reliance on dataset-specific artifacts or shortcuts. Furthermore, the classification report is computed per-slice (197 test slices), not per-patient, and slices from the same patient are not independent observations.

### 4.10. Comparative Analysis with Other Methods

Table 4 compares CardioSynergyNet’s final model against Dice scores reported in the external literature, which are each obtained under a different training protocol and data split; under this indirect comparison, CardioSynergyNet achieves a numerically higher Dice score (0.9050) than the compared methods (0.7352–0.9015).

As these figures stem from separate publications without shared patient-level data, this comparison represents a favorable point-estimate rather than a statistically verified improvement. This must be distinguished from controlled, same-conditions benchmark analysis. Under matched conditions, a single-task U-Net trained solely for segmentation achieves a significantly higher Dice score than CardioSynergyNet (0.8036 vs. 0.7594; *p* < 0.001, as shown in statistical significance analysis).

I do not claim that CardioSynergyNet’s segmentation accuracy exceeds that of a well-tuned single-task baseline under matched conditions; its contribution instead lies in achieving competitive segmentation alongside classification and biomarker estimation within a single jointly-trained model, which is a combination the single-task baseline cannot provide.

### 4.11. Ablation Study

To evaluate the contribution of each proposed module, an ablation study is conducted by systematically removing individual components from the full model, as summarized in Table 5. All ablation variants were trained for 40 epochs to reduce computational cost.

The ablation (Table 5) and baseline-comparison experiments reported in this and the following three subsections (Section 4.11, Section 4.12, Section 4.13 and Section 4.14) were conducted under a separate, shorter training protocol (40 epochs per variant) than the final model reported in Section 4.1, Section 4.2, Section 4.3, Section 4.4, Section 4.5, Section 4.6, Section 4.7, Section 4.8 and Section 4.9 (80 epochs, checkpoint at epoch 78). This choice was necessitated by computational constraints: the full training of CardioSynergyNet for 80 epochs required approximately 8 h on the GPU resource available (a Kaggle notebook session, capped at 12 h before automatic restart), which made repeated full-length training of the eight additional model variants required for the ablation study and baseline comparisons computationally impractical within this environment. A reduced 40-epoch budget was therefore adopted for these controlled comparisons, allowing all variants to be trained and compared under identical, matched conditions within the available compute budget. Consequently, the absolute Dice, classification accuracy, and other metrics in Table 5, Table 6, Table 7 and Table 8 are systematically lower than the fully trained final model’s performance and should not be directly compared to the headline results in the Abstract, Section 4.2, or Table 4.

The full model, incorporating all three modules (CPDA, DBEL, and CCPF), achieved a Dice score of 0.7594 and the highest classification accuracy of 0.6400, along with a Pearson correlation of 0.6962 for biomarker estimation. Removing CPDA (no_cpda) reduced the parameter count substantially from 77.52 M to 44.47 M but led to a noticeable drop in classification performance (Acc: 0.5900, F1: 0.5248) and segmentation boundary accuracy (HD95: 19.33 mm), indicating that CPDA plays a key role in refining spatial precision and class discrimination.

Excluding DBEL (no_dbel) yielded the best Dice score (0.7853) and lowest HD95 (11.69 mm) among all variants, and this difference was statistically significant at the patient level. This indicates that DBEL does not improve segmentation performance—rather, it comes at a measurable and statistically confirmed cost to segmentation accuracy. This cost is the price of enabling biomarker estimation, which is a capability that is entirely absent when DBEL is removed. DBEL should therefore be understood as necessary for the biomarker-estimation task specifically rather than as a component that improves the overall model performance.

The removal of CCPF (no_ccpf) resulted in the most significant performance degradation across nearly all metrics, with Dice dropping to 0.6516 and HD95 increasing sharply to 27.40 mm, while still maintaining reasonable biomarker correlation (0.6857). These results collectively demonstrate that each module contributes distinct and complementary benefits with CCPF proving most critical for overall segmentation quality and CPDA and DBEL each playing specialized roles in classification performance and biomarker estimation, respectively.

### 4.12. Comparison with Baseline Models

Table 6 compares CardioSynergyNet against two baselines: a single-task U-Net trained solely for segmentation and a vanilla multi-task learning (MTL) model with a shared encoder. As expected, the segmentation-only U-Net achieved the highest Dice score (0.8036) and lowest HD95 (6.1576 mm), since it dedicates its entire capacity to a single objective without the representational trade-offs inherent to multi-task learning. However, this baseline is architecturally incapable of performing classification or biomarker estimation, limiting its clinical utility to segmentation alone.

These results indicate that CardioSynergyNet achieves segmentation performance statistically indistinguishable from the vanilla shared-encoder baseline (Table 8), while showing numerically higher classification accuracy (0.6350 vs. 0.5270) and F1-score (0.5796 vs. 0.4835) together with the unique addition of biomarker estimation. However, patient-level significance testing (Table 8) shows that this classification difference does not reach statistical significance at the current sample size (n = 20 patients). I present this as a numerical improvement in classification performance, together with an added biomarker-estimation capability absent from the baseline, rather than as a statistically established advantage of the proposed fusion mechanisms over a naive shared-encoder approach. A larger patient cohort would be needed to determine whether this numerical difference reflects a true effect.

Notably, CardioSynergyNet also reduces the end-systolic HD95 for the right ventricle (ES_HD95_RV) from 28.0722 mm to 25.6594 mm relative to the vanilla MTL model, indicating improved boundary delineation during the more challenging end-systolic phase despite the added task complexity. Furthermore, CardioSynergyNet is the only model capable of jointly estimating clinical biomarkers, achieving a mean absolute error of 1.6748 and a Pearson correlation of 0.6407, which is a capability entirely absent in both baselines.

### 4.13. Efficiency Profile

Table 7 reports the computational efficiency of each model at inference time using a batch size of 1. CardioSynergyNet requires more parameters (77.52 M) and FLOPs (295.353 G) than both baselines, resulting in a higher mean latency of 80.621 ms compared to 45.623 ms for U-Net and 45.616 ms for SharedEncoderMTL. Peak memory usage also increases slightly from approximately 2791–2815 MB in the baselines to 2915.912 MB in CardioSynergyNet. This increase in computational cost is expected given the additional modules required for multi-task learning and biomarker estimation, and it remains within a practical range for deployment, since the added latency (roughly 35 ms) is negligible for most clinical workflows that do not require real-time inference.

### 4.14. Statistical Significance Testing

Table 8 presents paired statistical tests (*t*-test and permutation test) comparing CardioSynergyNet against baselines and ablation variants across 20 patients. CardioSynergyNet’s Dice score is significantly lower than U-Net (*p* < 0.001) and no_dbel (*p* < 0.001), but it shows no significant difference from SharedEncoderMTL or no_cpda, indicating that segmentation performance is largely preserved despite added task complexity. Notably, CardioSynergyNet significantly outperforms no_ccpf (*p* < 0.001) and both CCPF iteration variants (*p* < 0.01), confirming that the CCPF module meaningfully contributes to segmentation quality. For classification accuracy (correct), no comparisons reach statistical significance, suggesting that while CardioSynergyNet shows numerically higher classification performance than several variants, these differences are not statistically robust at the current sample size.

### 4.15. Physiological Plausibility of Deformation Fields

To evaluate physiological plausibility, I conducted a Jacobian determinant (det(J)) analysis on the deformation fields generated by the trained CPDA module across the test set. The empirical statistics confirm strict area-preserving and topologically sound behavior:

1. Topological Integrity: The mean folding rate (det(J)<0) across test batches was 0.000%, confirming the absence of self-intersections or spatial grid inversions.
2. Area-Preserving Behavior: The mean Jacobian determinant was 0.9982 (median: 0.9983, standard deviation: 0.0345), with the 1st and 99th percentiles tightly bounded between 0.9100 and 1.0903, where det(J)=1.0 represents perfect area preservation.

These quantitative results demonstrate that the learned CPDA transformations remain strictly smooth, bounded, and physiologically plausible without requiring additional explicit biomechanical regularization penalties during training.

## 5. Conclusions

This paper presented CardioSynergyNet, which is a single network that jointly performs cardiac MRI segmentation, biomarker estimation, and disease classification by linking the three tasks internally rather than combining separate models. Evaluated on 150 patients across five diagnostic groups, the model achieved a mean test Dice score of 0.9050 (computed across all four classes, including background) with the expected ED-over-ES advantage and the right ventricle as the hardest structure to segment. Biomarker regression was strong for area and mass-based quantities (R > 0.92) but weaker for ratio-based biomarkers, since small errors in near-zero denominators are amplified disproportionately. While the overall disease classification accuracy reached 76.65%, this confusion pattern is consistent with genuine clinical overlap in the imaging appearance of these conditions, though it does not, by itself, establish that the model’s classification decisions are driven by anatomically meaningful features rather than dataset-specific artifacts or shortcuts. Confirming this would require dedicated interpretability analyses, such as saliency methods validated against expert-identified regions, or evaluation on an external, independently acquired dataset.

Study limitations include the modest dataset size and reduced reliability for ratio-based biomarkers. The model also struggled with right ventricle segmentation and infarction classification. Furthermore, classification metrics were reported at the slice level without patient-level aggregation, such as majority voting, despite slices from the same patient lacking statistical independence. Incorporating the DBEL module introduced a trade-off by significantly reducing segmentation accuracy. Lastly, GPU time constraints limited ablation and baseline experiments to a 40-epoch training budget. As a result, these comparisons reflect relative module effects rather than absolute final performance, and future work with greater resources should re-evaluate them at full convergence.

## Figures and Tables

**Table 1:** Per-class Dice scores on the test set separated by cardiac phase.

Structure	ED Dice	ES Dice
Background	0.9977	0.9975
Right Ventricle (RV)	0.8542	0.6931
Myocardium (Myo)	0.8437	0.8291
Left Ventricle (LV)	0.9266	0.8522
Mean (all classes, including background)	0.9055	0.8430

**Table 2:** Mean Dice score per disease group and cardiac phase.

Disease Group	ED Dice	ES Dice
Abnormal Right Ventricle	0.8789	0.8252
Dilated Cardiomyopathy	0.8807	0.8011
Hypertrophic Cardiomyopathy	0.8910	0.7493
Myocardial Infarction	0.8462	0.7740
Normal	0.8855	0.7913

**Table 3:** Biomarker prediction performance on the test set. MAE is reported in the biomarker’s normalized units (log-transformed for the RV/LV ratio); R is the Pearson correlation coefficient.

Biomarker	MAE	Correlation (R)
EF proxy	0.152	0.806
Myocardial mass proxy	0.119	0.950
log(RV/LV) ratio (ED)	2.245	0.336
Wall thickness (ED)	0.104	0.878
LV area (ED)	0.264	0.968
LV area (ES)	0.140	0.981
RV area (ED)	0.184	0.981
RV area (ES)	0.101	0.919

**Table 4:** Performance comparison between CardioSynergyNet and existing models.

Reference	Model	Dice Score
[33]	GAN	0.7352
[34]	MAE-TransRNet	0.812
[35]	3D U-Net	0.884
[36]	FE-SwinUper	0.9015
[37]	Semi-supervised Attention U-Net	0.8934
Proposed	CardioSynergyNet	0.9050

**Table 5:** All models were trained for 40 epochs under the controlled ablation protocol.

Variant	Included Modules	Params	Dice Mean	HD95 Mean	Cls Acc	Cls F1	Biomarker MAE	Biomarker Pearson r
Full Model	CPDA + DBEL + CCPF	77.52 M	0.7594	15.11 mm	0.6400	0.5968	1.6769	0.6962
no_cpda	DBEL + CCPF	44.47 M	0.7476	19.33 mm	0.5900	0.5248	1.5050	0.6242
no_dbel	CPDA + CCPF	76.66 M	0.7853	11.69 mm	0.5910	0.5705	N/A*	N/A *
no_ccpf	CPDA + DBEL	77.49 M	0.6516	27.40 mm	0.6050	0.5589	1.2331	0.6857

* N/A: Not applicable/available for this variant.

**Table 6:** All models were trained for 40 epochs under the controlled baseline protocol.

Model	Params	Dice Mean	HD95 Mean	ASD Mean	ES HD95 (RV)	Cls Acc	Cls F1	Biomarker MAE	Biomarker r
UNetBaseline (seg-only)	31.04 M	0.8036	6.1576	2.0506	10.2637	–	–	–	–
SharedEncoderMTL (vanilla MTL)	43.56 M	0.7601	15.4851	4.3176	28.0722	0.5270	0.4835	–	–
CardioSynergyNet (full, ours)	77.52 M	0.7594	17.2107	4.6540	25.6594	0.6350	0.5796	1.6748	0.6407

**Table 7:** Efficiency profile (Batch Size 1, Single Forward Pass).

Model	Params	Params (M)	FLOPs (G)	Latency Mean (ms)	Latency Std (ms)	Peak Mem (MB)
UNetBaseline	31,036,676	31.037	167.050	45.623	0.732	2791.232
SharedEncoderMTL	43,557,325	43.557	167.051	45.616	0.432	2814.670
CardioSynergyNet (ours)	77,516,816	77.517	295.353	80.621	0.750	2915.912

**Table 8:** Statistical significance testing: CardioSynergyNet vs. baselines/ablations.

Comparison	Metric	N	Mean A	Mean B	Mean Diff	CI Low	CI High	t-Stat	p (*t*-Test)	p (Perm)	Sig.
CardioSynergyNet vs. UNetBaseline	Dice Mean	20	0.7541	0.8078	−0.0537	−0.0830	−0.0283	−3.8310	0.0011	0.0003	***
CardioSynergyNet vs. SharedEncoderMTL	Dice Mean	20	0.7541	0.7633	−0.0092	−0.0314	0.0090	−0.8867	0.3863	0.4442	n.s.
CardioSynergyNet vs. SharedEncoderMTL	Correct	20	0.6009	0.4936	0.1073	−0.0151	0.2363	1.6203	0.1217	0.1252	n.s.
CardioSynergyNet vs. no_cpda	Dice Mean	20	0.7541	0.7473	0.0068	−0.0067	0.0191	1.0065	0.3268	0.3331	n.s.
CardioSynergyNet vs. no_cpda	Correct	20	0.6009	0.5451	0.0558	−0.0323	0.1483	1.1757	0.2542	0.2672	n.s.
CardioSynergyNet vs. no_dbel	Dice Mean	20	0.7541	0.7877	−0.0337	−0.0492	−0.0196	−4.3458	0.0003	0.0001	***
CardioSynergyNet vs. no_dbel	Correct	20	0.6009	0.5939	0.0069	−0.0979	0.1113	0.1277	0.8997	0.9036	n.s.
CardioSynergyNet vs. no_ccpf	Dice Mean	20	0.7541	0.6585	0.0956	0.0760	0.1149	9.5287	0.0000	0.0000	***
CardioSynergyNet vs. no_ccpf	Correct	20	0.6009	0.5779	0.0229	−0.0385	0.0850	0.7135	0.4842	0.4974	n.s.
CardioSynergyNet vs. ccpf_iter_2	Dice Mean	20	0.7541	0.7198	0.0342	0.0151	0.0579	3.0510	0.0066	0.0012	**
CardioSynergyNet vs. ccpf_iter_2	Correct	20	0.6009	0.6295	−0.0287	−0.1289	0.0748	−0.5319	0.6010	0.6046	n.s.
CardioSynergyNet vs. ccpf_iter_3	Dice Mean	20	0.7541	0.7135	0.0406	0.0147	0.0695	2.8778	0.0096	0.0039	**
CardioSynergyNet vs. ccpf_iter_3	Correct	20	0.6009	0.6629	−0.0620	−0.1551	0.0347	−1.2632	0.2218	0.2305	n.s.

**Note:** *** p<0.001, ** p<0.01, n.s. = not significant p>0.05.

## Data Availability

The dataset analyzed in this paper is publicly available in the Kaggle repository under the Automated Cardiac Diagnosis Challenge (ACDC) at https://www.kaggle.com/datasets/samdazel/automated-cardiac-diagnosis-challenge-miccai17 accessed on March 2026. A comprehensive description of the dataset, including image acquisition protocols and benchmark details, is available in the foundational publication at https://doi.org/10.1109/TMI.2018.2837502 accessed on March 2026.

## Supplementary Materials

The following supporting information can be downloaded at https://www.mdpi.com/article/10.3390/tomography12090137/s1, Figure S1: Success case (patient099, Abnormal Right Ventricle). Segmentation Dice = 0.930; predicted disease class matched the true class (Abnormal Right Ventricle). Both segmentation and classification succeed for this case, illustrating the model’s typical behavior on a well-conditioned slice with clearly visible right ventricle, myocardium, and left ventricle; Figure S2: Failure case (patient001, Dilated Cardiomyopathy). Segmentation Dice = 0.000, computed on a slice where the ground-truth mask contains no foreground labels (an apical/basal slice outside the annotated cardiac extent); the model nonetheless predicted right ventricle, myocardium, and left ventricle regions, driving Dice to zero despite the disease classification remaining correct. This illustrates that a Dice score of zero at the slice level does not necessarily indicate a diagnostically meaningful segmentation failure, and that segmentation and classification errors do not always co-occur; Figure S3: Failure case (patient141, Abnormal Right Ventricle). Segmentation Dice = 0.000 under the same noforeground- ground-truth condition as Figure S2; the model again predicted plausible-looking structures on a slice with no annotated anatomy, while the disease classification remained correct (Abnormal Right Ventricle). This case is included alongside Figure S2 to show that this divergence pattern recurs across different disease classes; Figure S4: Failure case (patient147, Abnormal Right Ventricle, predicted Myocardial Infarction). Segmentation Dice = 0.000 on a slice with genuinely small, low-contrast right ventricle and myocardium regions; the model produced a spurious, mislocalized left-ventricle-labeled prediction and did not recover the true structures. The disease classification was also incorrect (predicted Myocardial Infarction). This is an example of a case where segmentation and classification failures co-occur, plausibly because both tasks are undermined by the same underlying difficulty: poor tissue contrast and very small structure size; Figure S5: Failure case (patient047, Myocardial Infarction, predicted Hypertrophic Cardiomyopathy). Segmentation Dice = 0.000 on a near-empty basal/apical ground-truth slice; the model produced a small, spurious right-ventriclelabeled prediction not present in the ground truth. Disease classification was also incorrect (predicted Hypertrophic Cardiomyopathy). As in Figure S4, this case shows segmentation and classification errors occurring together on a difficult, low-information slice; Figure S6: Failure case (patient040, Hypertrophic Cardiomyopathy, predicted Myocardial Infarction). Segmentation Dice = 0.951, among the highest in the test set, with predicted right ventricle, myocardium, and left ventricle boundaries closely matching the ground truth at both ED and ES. Despite this, the disease classification was incorrect (predicted Myocardial Infarction). This case is the clearest example in this cohort of the two tasks diverging: near-perfect anatomical segmentation coexisting with an incorrect diagnostic prediction, indicating that accurate structural delineation does not guarantee correct downstream classification when the disease-discriminating cues lie beyond gross chamber and wall geometry.
