# Supplementary material for: CardioSynergyNet: A Closed-Loop Multi-Task Deep Learning Architecture for Cardiac Segmentation and Biomarkers with Diagnosis from Paired ED-ES Cine-MRI
Source: Tomography. 2026 Sep 21;12(9):137. doi: 10.3390/tomography12090137 (PMC13611576; doi:10.3390/tomography12090137)
Supplement: Supplementary file 1 [file tomography-12-00137-s001.zip › tomography-4446565-supplementary.pdf]

Supplementary Materials

CardioSynergyNet: A Closed-Loop Multi-Task Deep Learning Architecture for Cardiac Segmentation and Biomarkers with Diagnosis from Paired ED-ES Cine-MRI

Qualitative Success and Failure Case Analysis. The following six figures (Figures S1–S6) present representative slice-level cases from the held-out test set, selected to illustrate the range of agreement and disagreement between the segmentation and disease-classification outputs of CardioSynergyNet. Each figure shows, for a single patient, the end-diastolic (ED) and end-systolic (ES) input images (left column), the ground-truth segmentation overlay (centre column), and the model's predicted segmentation overlay (right column); colour coding follows the main text (red = right ventricle, green = myocardium, blue = left ventricle). The per-slice Dice score and the true versus predicted disease class are reported in each figure title. Figures S1 and S6 show cases of accurate segmentation, with S1 additionally showing correct classification and S6 showing a classification error despite near-perfect segmentation. Figures S2 and S3 show cases in which segmentation Dice is zero due to the absence of annotated foreground in the ground-truth slice, despite the disease classification remaining correct. Figures S4 and S5 show cases in which both segmentation and classification fail concurrently, on slices with small, low-contrast cardiac structures. Taken together, these cases indicate that segmentation and classification errors in CardioSynergyNet are only partially coupled: each task can fail independently of the other, and neither task's accuracy is a reliable proxy for the other's on a given slice.

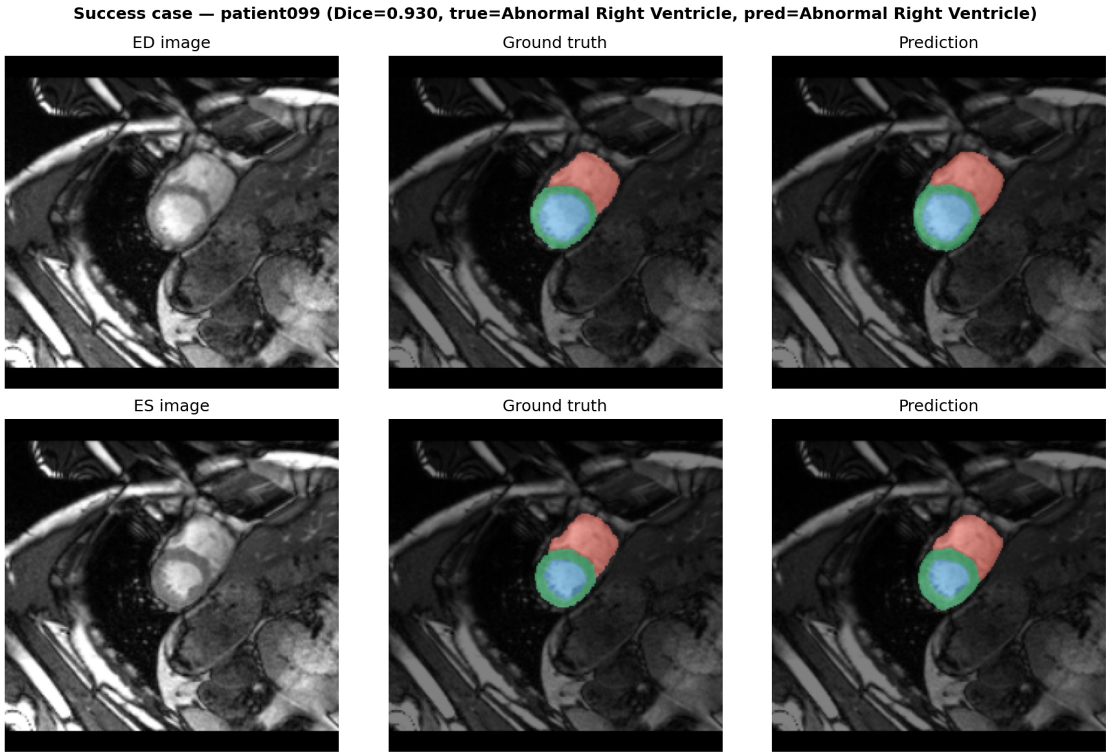

Figure S1. Success case (patient099, Abnormal Right Ventricle). Segmentation Dice = 0.930; predicted disease class matched the true class (Abnormal Right Ventricle). Both segmentation and classification succeed for this case, illustrating the model's typical behavior on a well-conditioned slice with clearly visible right ventricle, myocardium, and left ventricle.

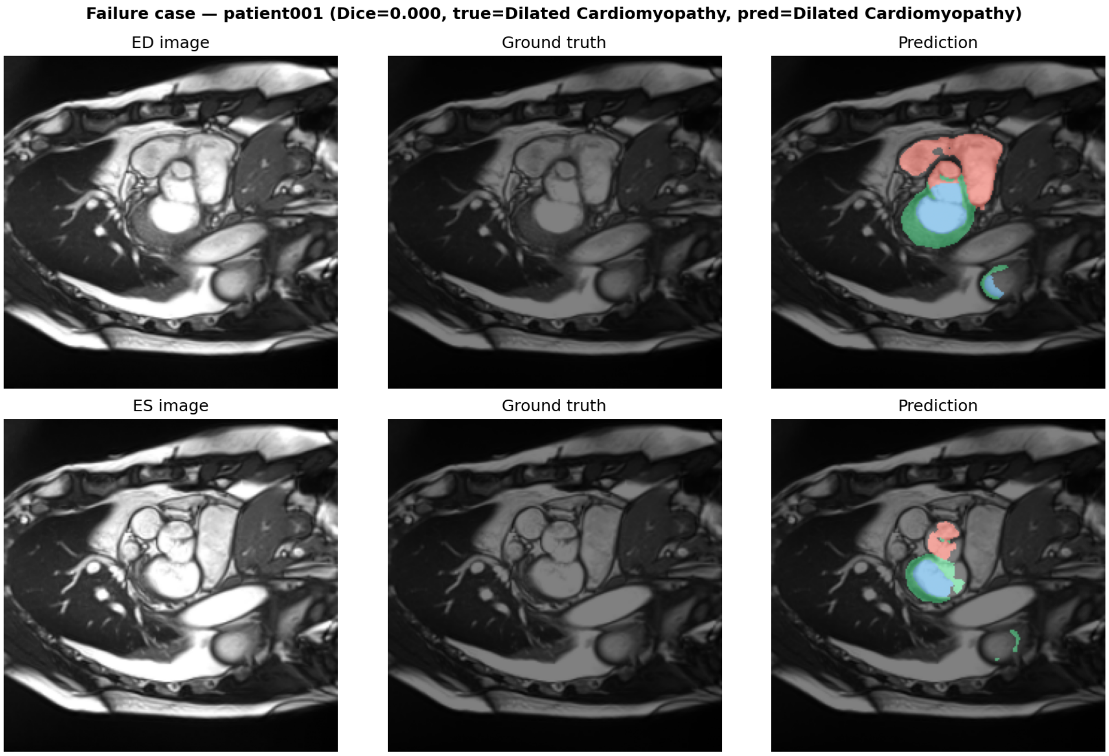

Figure S2. Failure case (patient001, Dilated Cardiomyopathy). Segmentation Dice = 0.000, computed on a slice where the ground-truth mask contains no foreground labels (an apical/basal slice outside the annotated cardiac extent); the model nonetheless predicted right ventricle, myocardium, and left ventricle regions, driving Dice to zero despite the disease classification remaining correct. This illustrates that a Dice score of zero at the slice level does not necessarily indicate a diagnostically meaningful segmentation failure, and that segmentation and classification errors do not always co-occur.

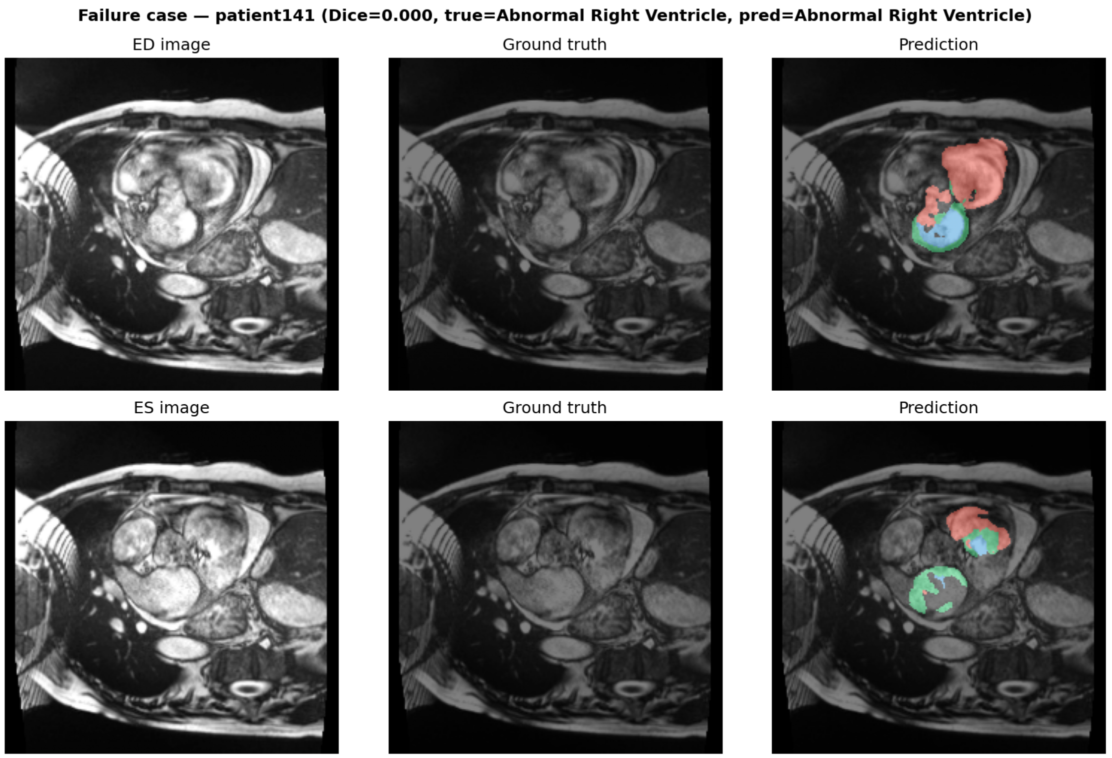

Figure S3. Failure case (patient141, Abnormal Right Ventricle). Segmentation Dice = 0.000 under the same no-foreground-ground-truth condition as Figure S2; the model again predicted plausible-looking structures on a slice with no annotated anatomy, while the disease classification remained correct (Abnormal Right Ventricle). This case is included alongside Figure S2 to show that this divergence pattern recurs across different disease classes.

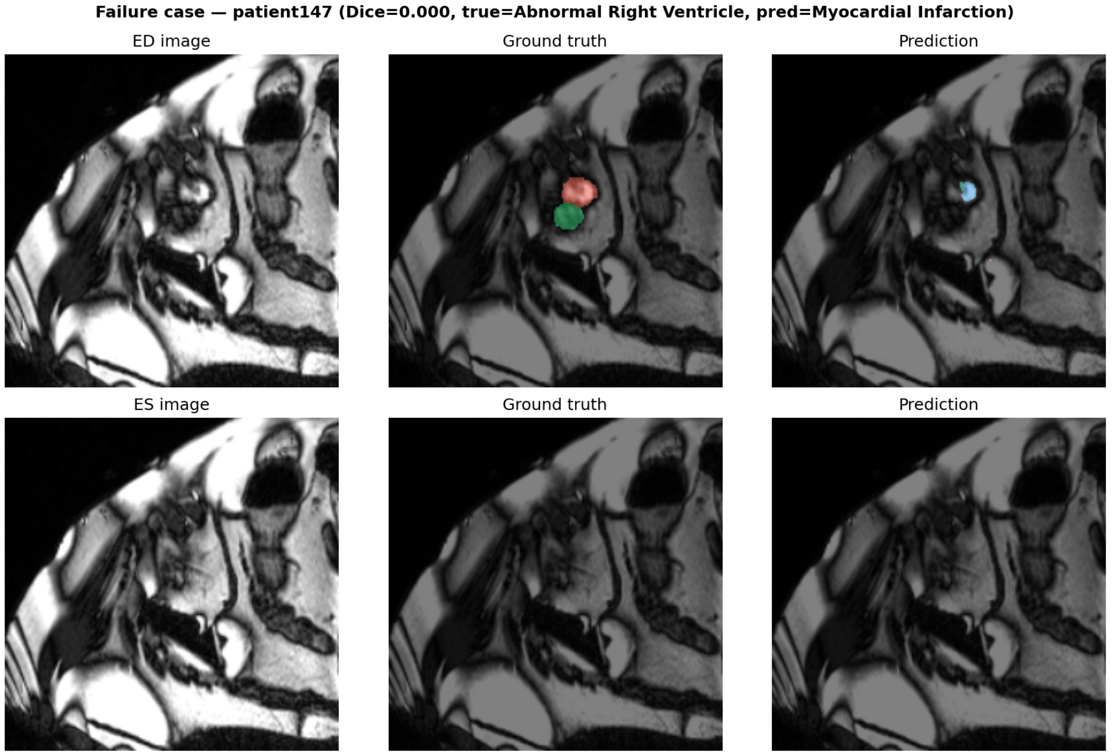

Figure S4. Failure case (patient147, Abnormal Right Ventricle, predicted Myocardial Infarction). Segmentation Dice = 0.000 on a slice with genuinely small, low-contrast right ventricle and myocardium regions; the model produced a spurious, mislocalized left-ventricle-labeled prediction and did not recover the true structures. The disease classification was also incorrect (predicted Myocardial Infarction). This is an example of a case where segmentation and classification failures co-occur, plausibly because both tasks are undermined by the same underlying difficulty: poor tissue contrast and very small structure size.

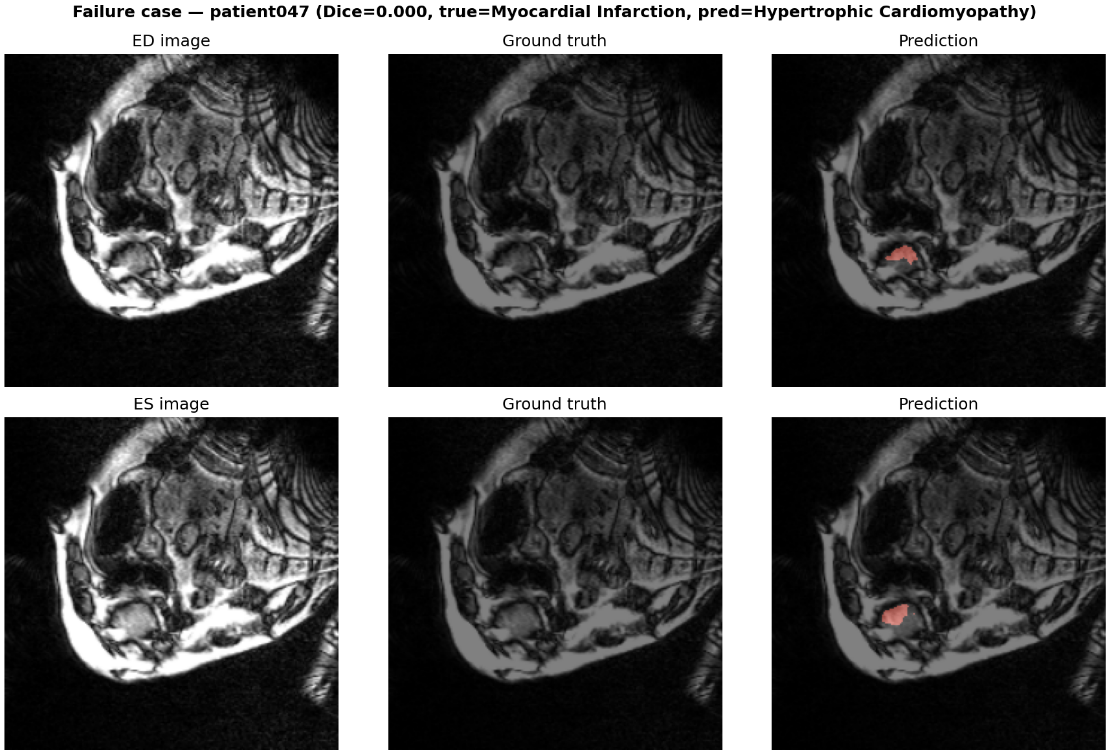

Figure S5. Failure case (patient047, Myocardial Infarction, predicted Hypertrophic Cardiomyopathy). Segmentation Dice = 0.000 on a near-empty basal/apical ground-truth slice; the model produced a small, spurious right-ventricle-labeled prediction not present in the ground truth. Disease classification was also incorrect (predicted Hypertrophic Cardiomyopathy). As in Figure S4, this case shows segmentation and classification errors occurring together on a difficult, low-information slice.

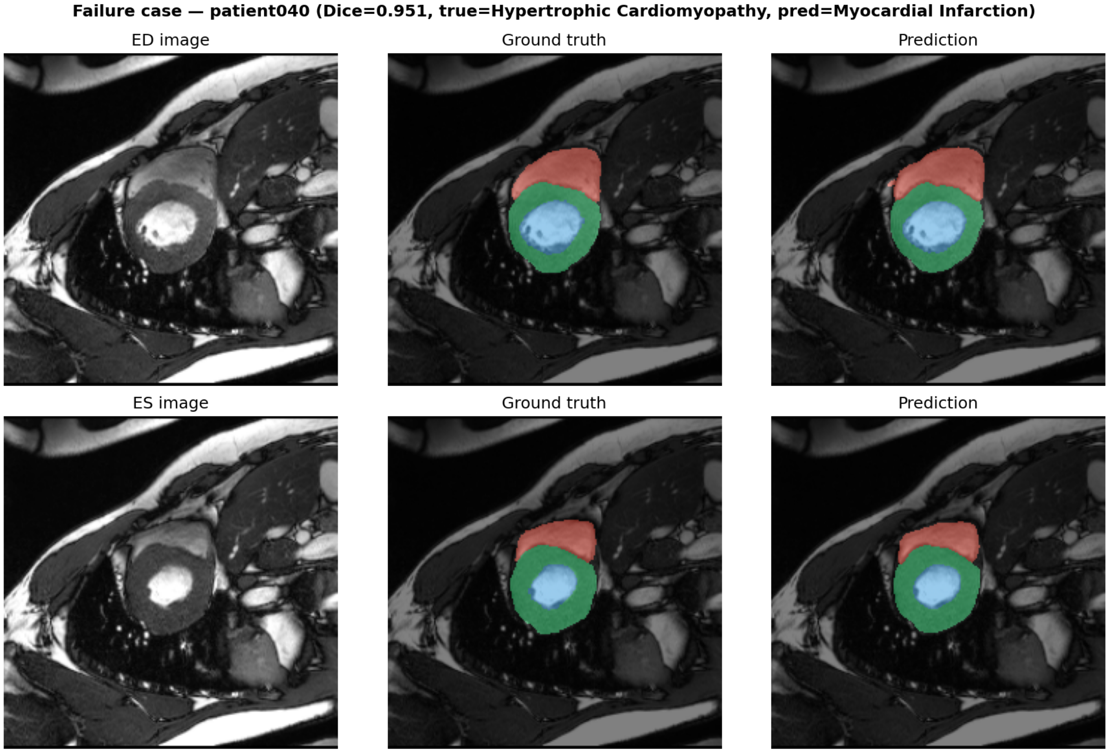

Figure S6. Failure case (patient040, Hypertrophic Cardiomyopathy, predicted Myocardial Infarction). Segmentation Dice = 0.951, among the highest in the test set, with predicted right ventricle, myocardium, and left ventricle boundaries closely matching the ground truth at both ED and ES. Despite this, the disease classification was incorrect (predicted Myocardial Infarction). This case is the clearest example in this cohort of the two tasks diverging: near-perfect anatomical segmentation coexisting with an incorrect diagnostic prediction, indicating that accurate structural delineation does not guarantee correct downstream classification when the disease-discriminating cues lie beyond gross chamber and wall geometry.
